# Differential induction of defense genes in hexaploid wheat roots by the plant-parasitic nematodes *Pratylenchus neglectus* and *P*. *thornei*

**DOI:** 10.1371/journal.pone.0306533

**Published:** 2024-08-29

**Authors:** Patricia A. Okubara, Richard M. Sharpe, Amy B. Peetz, Xianran Li, Inga A. Zasada

**Affiliations:** 1 Wheat Health, Genetics and Quality Research Unit, USDA-ARS, Pullman, Washington, United States of America; 2 Department of Horticulture, Washington State University, Pullman, Washington, United States of America; 3 Horticultural Crops Disease and Pest Management Research Unit, USDA-ARS, Corvallis, Oregon, United States of America; University of Limpopo, SOUTH AFRICA

## Abstract

*Pratylenchus neglectus* and *P*. *thornei* are among the most destructive root lesion nematodes of wheat in the Pacific Northwest, United States of America and throughout the world. The aim of this study was to determine whether both nematode species were similar in their ability to induce defense genes in roots of wheat genotype Scarlet, and whether a combination of both species induced a different pattern of gene induction than each species alone. The long-term aspect of the research was to identify nematode-inducible promoters for deploying defense genes in roots in breeding programs. The root transcriptomes of genotype Scarlet were obtained after a one-week infection period with each nematode species separately, or both species combined. Root defense gene expression was induced for all three treatments relative to the no-nematode control, but *P*. *thornei* affected expression to a greater extent compared to *P*. *neglectus*. The species combination induced the highest number of defense genes. This result was not predicted from nematode enumeration studies, in which *P*. *thornei* colonization was substantially lower than that of *P*. *neglectus*, and the nematode combination did not show a significant difference. Quantitative real time polymerase chain reaction (qRT-PCR) assays for *Dehydrin2*, *Glucan endo-1*,*3-beta-glucosidase*, *1-cys-Peroxiredoxin*, *Pathogenesis-related protein 1* and *Late embryogenesis-abundant proteins 76* and *group 3* authenticated the induction observed in the transcriptome data. In addition, a near-isogenic line of Scarlet harboring genetic resistance to fungal soilborne pathogens, called Scarlet-Rz1, showed similar or higher levels of defense gene expression compared to fungus-susceptible Scarlet in qRT-PCR assays. Finally, transcriptome expression patterns revealed nematode-inducible promoters that are responsive to both *P*. *neglectus* and *P*. *thornei*.

## Introduction

Hexaploid (bread) wheat (*Triticum aestivum* L.) is a major staple crop grown in the Pacific Northwest (PNW; Idaho, Oregon, Washington) of the United States, where over a million hectares of land are under wheat production. Wheat plays an important role in rotational schemes in the region [1]. Like most crops, wheat production is limited by plant-parasitic nematodes, including the root lesion nematodes (*Pratylenchus* spp.) [2]. *Pratylenchus neglectus* and *P*. *thornei* are problems in wheat-growing regions of the PNW and throughout the world [3,4]. Approximately 60% of dryland wheat fields in eastern Washington are infested with *P*. *neglectus* or *P*. *thornei* [5]. This level of infestation comes at a cost of over $50 million in reduced production of wheat [3]. While *P*. *neglectus* is more prevalent in the region than *P*. *thornei*, mixed populations are also common [6]. The damage to wheat caused by these nematodes is dependent on many variables, including time of year when wheat is grown (spring vs. winter), temperature, wheat variety (genotype), and soil abiotic factors. In addition, higher yield losses by *P*. *thornei* have been reported relative to *P*. *neglectus*, with up to 50% and 35% for *P*. *thornei* and *P*. *neglectus*, respectively [7,8]. The most widely used management practices control of these nematodes are genetic resistance and crop rotation, e.g., [4]. Because of the significant impact of *Pratylenchus* spp. on the production of wheat, and the difficulty of controlling these nematodes with crop rotation due to their wide host ranges [9], new ways to manage these nematodes are needed.

Partial genetic resistance conferred by quantitative trait loci (QTL) has been identified in a variety of wheat genotypes, including diploid and tetraploid relatives of hexaploid wheat [10], recombinant inbred lines, doubled haploid lines [11] and Iranian land races [12,13]. Identification of defense genes within the mapped resistance loci has also been reported. Seven potential resistance gene candidates were identified in a genome-wide association mapping project involving 143 wheat genotypes segregating for resistance to *P*. *thornei* [14]. Other potential *Pratylenchus*-associated defense genes have been revealed by comparing defense gene expression in susceptible and resistant wheat cultivars. In two such studies, a *P*. *thornei*-resistant wheat cultivar displayed greater constitutive and induced accumulation of prenylpropanoids and higher polyphenol oxidase and peroxidase activities, compared to a susceptible cultivar [15]. In one case, population densities of *P*. *thornei* also were lower in washed roots and roots with rhizosphere soil from the resistant cultivar, correlating increased phenylpropanoids and decreased parasitism with disease resistance. A similar result was reported earlier for phenylpropanoid pathway genes in alfalfa [16].

As a part of this transcriptome study, we examined *Pratylenchus*-mediated induction of six specific wheat root defense genes: *Late embryogenesis-abundant proteins 76* and *group 3*, *Dehydrin2*, *Lichenase* (a β-1,3/1,4-glucanase), *Pathogenesis-related protein 1* and *1-Cys peroxiredoxin*. The late embryogenesis-abundant (LEA) proteins are present in a broad range of developmental stages, organs and subcellular locations in plants [17], and confer protection against abiotic stresses, including heat, cold/freezing, heavy metal, drought and salinity [18]. The LEAs comprise eight structurally distinct protein groups. In potato roots, LEA protein family members were differentially expressed under drought and cold (4°C) but not heat (35°C) stress [18]. Proposed mechanisms of LEA-mediated stress protection include stabilization of membrane proteins, prevention of protein aggregation and other detrimental molecular interactions within the cell, and direct interaction with cellular enzymes such as glutathione S-transferase for promoting reactive oxygen species (ROS) detoxification [17]. LEA group 3 proteins protect phosphofructose kinase and mitochrondrial citrate synthase during dessication [19]. Dehydrins are Group II LEAs that are induced in response to cold, drought, osmotic and salinity stress, and also can prevent accumulation of reactive oxygen species (reviewed in [20]). They are known to bind water and specific lipid components of cellular membranes, and by this mechanism are thought to protect the cell under conditions that impose cellular dehydration. In addition, protection against damage caused by freeze-thaw cycles has been attributed to direct binding of LEAs to lactate dehydrogenase and α-amylase [20].

Lichenase, named for its ability to break down the moss starch lichenin, is an endo-(1–3),(1–4)-β-D-glucanase [21]. Lichenase is one of three classes of glycosidases that act to destabilize microbial cell walls. The enzyme generates β-1,3/1,4-glucan oligosaccharides that induce pattern-triggered immunity in plants [21,22]. Lichenase and β-1,3- glucanases are evolutionarily related and portions of their sequences are indistinguishable [21]. The former is induced by wounding and by infection with the foliar fungal pathogen *Magnaporthe grisea* in rice [21], but induction by root pathogens such as *Pratylenchus* has not been reported to our knowledge. Pathogenesis-related protein 1 (PR-1) is among the earliest described pathogen-induced proteins in TMV-infected tobacco, in which pathogen attack is countered by a strong resistance response called the hypersensitive response. PR-1 is induced by the defense signal molecules salicylic acid and jasmonate [23]. The anti-microbial activity of PR-1 has not been attributed to an enzymatic or membrane-disrupting activity, such as β-1,3-glucanase (PR-2) or thaumatin-like protein (PR-5). PR-1 is included in the CAP superfamily (Cysteine-rich secretory proteins, Antigen 5, and Pathogenesis-related 1 proteins) and with Major birch pollen allergen Bet v 1, based on similarities in protein structure. The entire PR-1 protein consists of the CAP domain [24]. The PR-1 proteins are allergenic because they are small, stable, secreted from the cell and resistant to proteases [24]. Peroxiredoxins are part of the oxidative stress (ROS) metabolic pathway, acting through ascorbate or the glutathione redox cycle to reduce accumulation of H_2_O_2_ in the cell. The 1-cys-peroxiredoxin shares sequence similarity to the LEAs, and its barley orthologue was expressed late in seed development [25]. Overall, these selected proteins and their genes represent four different mechanisms of defense and stress tolerance in plants.

## Materials and methods

### Nematode culture, root treatments and enumeration

Nematode cultures were obtained from Dr. Richard Smiley, Oregon State University, Pendleton, OR, as described in Smiley et al. [9]. The populations of *Pratylenchus neglectus* and *P*. *thornei* were originally collected from wheat fields. *Pratylenchus neglectu*s inoculum was comprised of five isolates from infested fields in Oregon and Washington; *P*. *thornei* inoculum contained a single isolate from an Oregon field. Cultures of each species were derived from single surface-sterilized adult females grown on carrot disks and cultured under aseptic laboratory conditions at 22°C for at least 3 months. A molecular method based on the 28S rRNA D3 expansion domain was used to originally identify the *Pratylenchu*s species [26]. Upon transfer of cultures to the USDA-ARS in Corvallis, OR, the identity of the cultures was confirmed with a molecular method based on the β-1,4-endoglucanase gene [27]. The cultures were increased on carrot disks to generate inoculum and viewed under a microscope for vitality prior to inoculation of plants [28].

Individual plants of the *Rhizoctonia*-susceptible wheat cultivar Scarlet and the *Rhizoctonia*-resistant isogenic line Scarlet-Rz1 (Rz1) [29] were grown in 15 cm plastic cones (Stuewe and Sons, Corvallis, Oregon, United States of America) containing autoclaved sand. As Rz1 was slower to germinate, Rz1 seeds were placed on Petri plates 3–5 days earlier and sown into sand 1–2 days earlier than Scarlet, so that seedling emergence occurred at the same time. Seedlings were grown at 25°C, 16 h light/20°C, 8 h dk in a mist chamber. About 2000 mixed-stage individuals (juveniles and females) of each nematode species were introduced in 5 mL of water to roots of 14-day-old plants.

One experiment consisted of four treatments: 1—no-nematode control (Cont); 2—*Pratylenchus neglectus* only (Pn); 3—*P*. *thornei* only (Pt); and 4—a 1:1 mixture (4000 nematodes) of *P*. *neglectus* and *P*. *thornei* (PnPt or Pn+Pt). After a 7-day infection period, three roots from each treatment were rinsed, combined, and transferred to liquid nitrogen and stored at −80°C prior to RNA isolation. Population density estimates were obtained on pooled roots using intermittent mist for 5 days [30]. The experiment was conducted four times.

To assess the statistical significance (P < 0.05) of variation in nematode densities among the treatments and wheat genotypes, density data from all four experiments were analyzed using the general analysis of variance (ANOVA) and Fisher’s protected least significant difference (LSD) algorithm (Statistix 8.1 Analytical Software, St. Paul, Minnesota, United States of America). Roots from Experiment 1 were used to determine the efficiencies and optimal annealing temperatures of qRT-PCR assays, and those from Experiment 2 were used to generate the qRT-PCR gene expression results.

### Illumina RNA-seq

Total RNA extraction, transcriptome generation and analysis of raw sequence reads have been described in [31]. Briefly, RNA from three biological replicates of each of four nematode treatments (12 samples) were sequenced by MRDNA (Shallowater, Texas, United States of America). RNA quality was based on sizes of all RNA fragments obtained using the Bioanalyzer and the Agilent 2100 software [32]. Twelve sample libraries were prepared from 1.0 μg of total RNA using the TruSeq™ RNA LT Sample Preparation Kit (Illumina, San Diego, California, United Sates of America). Libraries were indexed using barcodes and adaptors and 2 nM of each library were pooled. The pooled mix consisting of 5 pM of each library was subjected to Illumina HiSeq 2500 paired-end 2×150 sequencing. The CLC Genomics Workbench Trim Sequences tools (Qiagen Bioinformatics, Redwood City, California, United States of America) were used to filter raw sequence reads for quality, and to remove adaptors, index barcodes, and ambiguous bases. *De novo* assembly of filtered reads into contiguous sequences (contigs) was conducted using the De Bruijn graphing algorithm [33]. Merged reads greater than 85 bp in length with an overlap of 20–22 bp were assembled into contiguous sequences (contigs) representing the expressed transcripts. Raw read files are available as NCBI BioProject ID PRJNA512537, accession numbers SAMN10686471-10686492.

### Annotation and transcriptome analysis

Transcript counts from triplicate samples were averaged for each treatment, and averages were normalized among treatments using reads per kilobase of transcript per million mapped reads (RPKM) [34]. Transcript contigs were initially identified using the International Wheat Genome Sequencing Consortium (IWGSC) coding sequence (transcriptome) database for *Triticum aestivum* [35], and gene model identifiers (gene IDs) were annotated using Blast2GO (BioBam Bioinformatics S.L., Valencia, Spain), as described in [31]. Gene IDs having RKPM values ≥51 when all four treatments were added together (total RPKM) were analyzed for induction or repression by nematodes. Fold-change ratios (*FC*, treated/control) were calculated using RPKM values for *Pn*, *Pt* and *Pn+Pt*. Log 2 values were calculated for each treatment using Excel, where *fx* = Log(*FC*,2). Gene IDs showing log2 fold-change values ≥1 were considered to be induced by *Pratylenchus* treatment, and those of ≤-1 repressed by treatment [31]. Coding sequences for this transcriptome-based set of gene IDs were used to generate primers for reverse transcription real-time PCR (qRT-PCR).

For this study, the above set of gene identifiers and their annotations were updated using the IWGSC genome RefSeq Annotation v1.0 [36], MIPSv2.2 [37], and the online tool *IDConverter* at the WheatOmics platform (http://wheatomics.sdau.edu.cn/idConvert/) [38]. Gene models were predicted with two independent pipelines previously utilized for wheat genome annotation and then consolidated to produce the RefSeq Annotation v1.0. Subsequently, a set of manually curated gene models was integrated to build RefSeq Annotation v1.1. In total, 107,891 high-confidence protein-coding loci were identified, with relatively equal distribution across the A, B, and D subgenomes. Partially supported gene models, gene fragments, and orphans were excluded from this set. A predicted function was assigned to 82.1% (90,919) of genes in RefSeq Annotation v1.0, and evidence for transcription was found for 85% (94,114) of the genes. From the TGACv1 annotation, 98,270 genes were aligned to the assembly using Exonerate [39]. For each gene, up to three alignments are displayed, comprising 196,243 alignments of which 90,686 are protein coding.

The mRNA and coding sequences of each gene ID were retrieved from the corresponding fasta files released in the IWGSC annotation (RefSeq v1.1) using samtools faidx [40]. The updated gene IDs were filtered to remove non-mappable contigs or gene IDs tagged as low confidence. Gene IDs having total RKPM values ≥51 were examined further to identify nematode-induced or -repressed transcripts, and those with potential roles in defense and stress. The updated genome-based gene IDs were used to support previous contig annotations, and to compare RPKM counts to qRT-PCR results. Gene IDs with defense/stress-related annotations were compiled in separate tables.

### Quantitative RT-PCR assays

Total RNA was prepared from roots of 21-day-old Scarlet and its near-isogenic line Rz1 following a 7-day infection period, as described in Nematode culture, root treatments and densities. RNA samples from two experiments were processed at the Center for Quantitative Life Sciences (Oregon State University, Corvallis, Oregon, United States of America) using the RNeasy Plant Mini Kit (Qiagen, Redwood City, California, United States of America).

Primers were designed from coding sequences of *Peroxiredoxin*, *Dehydrin2*, *Late embryogenesis-abundant* (LEA) *protein76*, *LEA protein group 3*, *Glucan endo-1*,*3-beta-glucosidase* (also called *beta-1*,*3-Glucanase* or *Lichenase*), and *Pathogenesis-related protein1*. The internal standard was *Translation elongation factor1-alpha*, or *TEF1*. Coding sequences having similar or identical annotations were aligned using Clustal Omega [41] to visualize regions of homogeneity. Potential gene-specific primers were analyzed for annealing temperature, % GC and formation of hairpins, self-dimers and heterodimers using Integrated DNA Technologies OligoAnalyzer^TM^ online tool. Blastn analyses was performed on candidate primers to look for non-target binding. Primers were purchased from Sigma-Aldrich Inc. (St. Louis, Missouri, United States of America).

Each cDNA synthesis consisted of 1 μg total RNA, 2.5 μM oligo (dT)_20_, 60 U of RNAse OUT (Invitrogen/Thermo Fisher Scientific, Waltham, Massachusetts, United States of America) and 300 U of Superscript III (Thermo Fisher Scientific) in a final volume of 30 μL. For standard curves, cDNA was diluted 4 times using a 1:5 serial dilution. Quantitative RT-PCR was performed on the StepOnePlus thermocycler (Applied Biosystems/Thermo Fisher Scientific). Each reaction contained 1 μL of cDNA and 1x GoTaq qPCR Master Mix (Promega Corp., Madison Wisconsin, United States of America), containing BRYT Green® Dye, in a total volume of 20 μL. Each sample was analyzed in triplicate. The amplification program was: 95°C 2 min, denaturation at 95°C 15 s, annealing for 15 s at the recommended temperatures shown in S1 Table, extension at 60°C 1 min for 55 cycles.

Relative fold-change values were calculated using the delta-delta C_t_ algorithm [42] and the TEF1 internal standard. Efficiency of PCR amplification was calculated using the equation E = 10^1/-m^ -1, where m is slope of the plot of log cDNA (x-axis) vs. cycle threshold (y-axis). Standard curve plots were generated from four to five 1:5 serial dilutions of cDNA from Experiment 1.

## Results and discussion

### Nematode population densities

**Fig 1:**
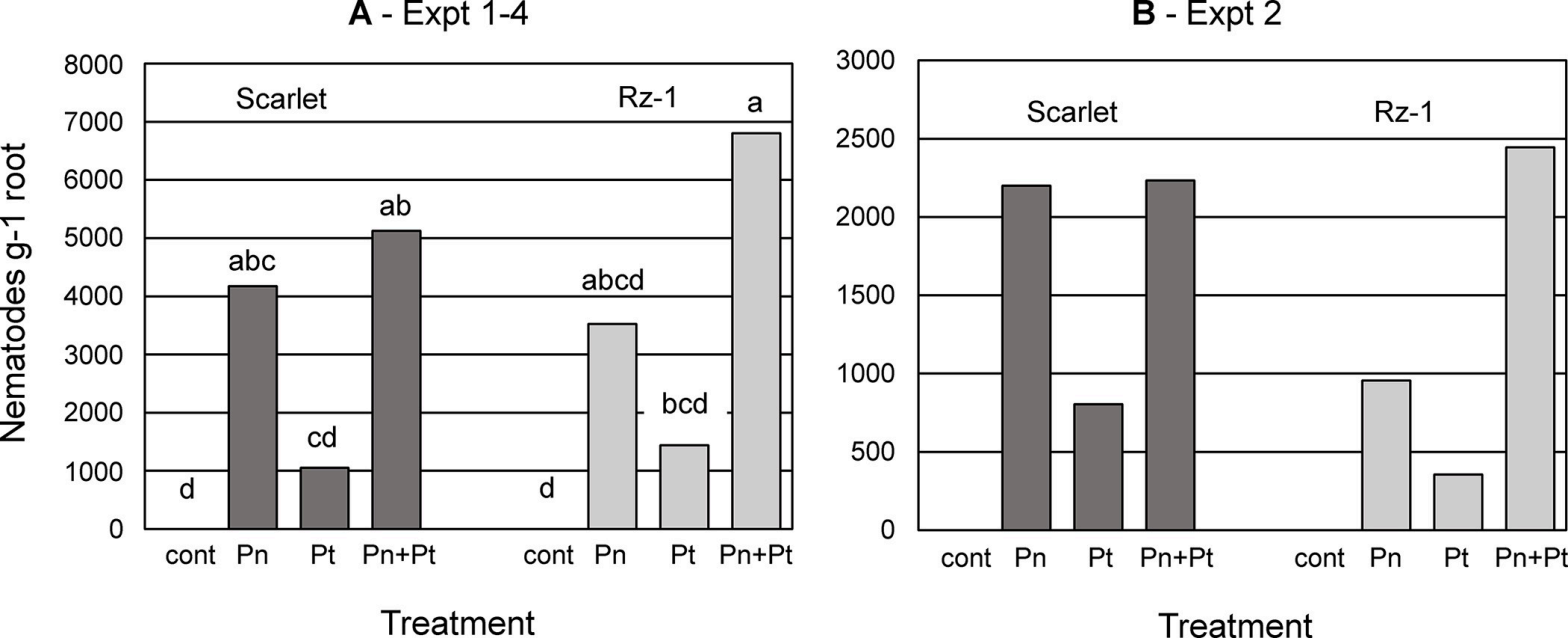
*Pratylenchus* population densities per gram root in Scarlet and Scarlet-Rz1. For each experiment, nematodes were extracted 7 days after inoculation from three 21-day-old plants, pooled prior to enumeration. A, Values are the average of four experiments. Letters indicate significant (*P* < 0.05) differences among means (Statistix, St. Paul, Minnesota, United States of America). B, Values from Experiment 2, which was used for qRT-PCR.

Two near-isogenic lines of hexaploid wheat, Scarlet and Scarlet-Rz1 (Rz1) [29], were used for this study. Data from four experiments (Fig 1A) provided biological replicates of each treatment for statistical analysis and showed that colonization of roots by *P*. *neglectus* was greater than that by *P*. *thornei* for both Scarlet and Rz1 genotypes. The population density of the *P*. *neglectus-P*. *thornei* combination in roots of Rz1 was slightly greater compared to Scarlet (Fig 1A), but the difference was not significant (P>0.05). Nematode population densities in roots of Experiment 2, used for qRT-PCR, showed very similar trends among treatments and wheat genotypes (Fig 1B), and Experiment 2 was representative of nematode infection. Population density data are shown in S7 Table.

### RNA libraries and contig annotations

Total RNA used for library construction ranged from 330–440 ng/μL for triplicate samples for each of four treatments, with RIN values of 4.1–5.3. The resulting libraries were composed of 30.6 ± 5.0 to 32.4 ± 0.5 ng uL^-1^ RNA representing average insert sizes of 485.3 ± 9.6 to 514.7 ± 20.2 bp [31].

Raw reads were assembled into contigs for annotation. Mapping of the contigs against the 293,058 coding sequences in the IWGSC database [36] resulted in 262,908 annotated Traes gene IDs; 218,150 of these displayed RPKM values >0 for each of the four treatments (S1A Table). A subset of 19,502 gene IDs with total RPKM values >51 were used for log2 fold-change calculations (S1B Table). Mapping of the contigs using the 269,583 IWGSC reference genome gene IDs [31] resulted in 84,396 Traes gene IDs having RPKM values >0 for each of the four treatments; 64,224 were assigned annotations at a high-confidence level (S2A Table). A subset of 64,224 gene IDs having total RPKM values >51 were used for log2 fold-change calculations (S2B Table) and other analyses. The genome-based dataset yielded fewer gene IDs than the coding sequence-based dataset, and the annotations in the former tended to be less descriptive. Since the second dataset was derived from the first, differences among the two datasets were not biologically significant, and the same highly-induced defense genes were identified in both sets.

### Induction and repression of root defense genes by *Pratylenchus*

A total of 3,256 defense- and stress-related gene IDs having total RPKM values ≥51, representing 522 annotation groups, were identified from the coding sequence dataset (S3A Table), and 2,615 defense/stress-related gene IDs (total RPKM ≥51) representing 466 annotation groups were identified from the genome-based dataset (S4A Table). These comprehensive collections of genes included gene IDs that were induced, repressed and non-differentially expressed. Coding sequence-based defense gene IDs that were induced or repressed by *P*. *neglectus*, *P*. *thornei* or the *P*. *neglectus*- *P*. *thornei* combination are shown in S3B-S3D Table, respectively. Induction or repression of the genome-based defense gene IDs by the three *Pratylenchus* treatments are shown in S3B-S3D Table, respectively. References for these annotation groups are listed in S5 Table. A total of 360 non-redundant annotation groups resulted when the two datasets were merged.

Table 1 shows the tallies of induced and repressed genes identified for each annotation dataset and treatment. Parasitism by *P*. *thornei* (Pt treatment) resulted in a greater number of induced gene IDs compared to *P*. *neglectus* (Pn treatment). The combined inoculation of *P*. *neglectus* and *P*. *thornei* (Pn+Pt treatment) yielded the most induced genes than either species alone. In the coding sequence-based dataset, the number of gene IDs in Pn+Pt treatment exceeded that of the sum of Pn and Pt. This might be due to the richer variation in gene annotation in the coding sequence database (compared to the wheat genome database) rather true synergistic effects on induction. The data suggest that both *Pratylenchus* species in combination contributed additively to gene induction. Although there were fewer repressed genes, a similar trend among treatments was observed.

**Table 1:** Tally of defense- and stress-related gene IDs in the coding sequence-based and genome-based datasets (total RPKM ≥51).

	Coding sequence-based dataset		Genome-based dataset	
Treatment	Induced	Repressed	Induced	Repressed
**Pn**	26	28	27	8
**Pt**	84	11	65	13
**Pn+Pt**	126	17	91	14

Overall, defense/stress gene IDs comprised 37%, 68% and 57% of all induced gene IDs in the coding sequence dataset for the Pn, Pt and Pn+Pt treatments, respectively. In the genome-based set, 59%, 74% and 62% were defense/stress-related for the Pn, Pt and Pn+Pt treatments, respectively. With the exception of the coding sequence-based gene IDs for *P*. *neglectus*, more genes were induced than repressed in the treatments.

**Fig 2:**
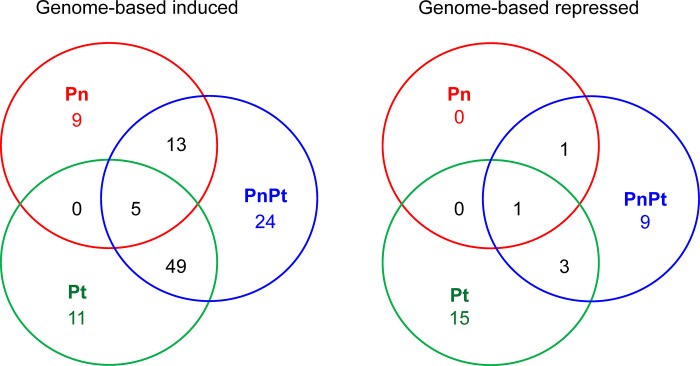
Venn diagrams of defense/stress-related genes induced (log2 fold-change ≥1.0) and repressed (log2 fold-change ≤-1.0) in wheat roots after *Pratylenchus neglectus* (Pn, red), *P*. *thornei* (Pt, green) and combined (Pn+Pt, blue) treatments. Values were obtained from genome-based gene IDs having RPKM values ≥51 when all four treatments were summed. Induced (log2 fold-change ≥1) and repressed (log2 fold-change ≤-1) defense/stress-related gene IDs for Pn, Pt and Pn+Pt treatments are listed in S4B-S4D Table, respectively.

A comparison of gene induction or repression by the *Pratylenchus* treatments was visualized for the genome-annotated dataset using Venn diagrams (Fig 2). More genes were induced than repressed for all *Pratylenchus* treatments. The Pn and Pt treatments shared 13 and 49 induced gene IDs with the Pn+Pt treatment, respectively. Twenty-four gene IDs were uniquely induced in the Pn+Pt combination; only 5 gene IDs were induced in all three *Pratylenchus* treatments. The repressed gene IDs were more treatment-specific. The Pt treatment resulted in 15 uniquely-repressed gene IDs, and only 1 gene ID was common among all the treatments. The results suggested that wheat roots respond differentially to *P*. *neglectus* and *P*. *thornei*, and that the latter species has a greater impact on defense gene expression.

To account for minor differences in annotation between the datasets, generic names were substituted. For instance, “late embryogenesis abundant protein-related / LEA protein-related LENGTH = 559” was simplified to “late embryogenesis abundant protein.” The two datasets were merged, resulting in 158 (43.9%) non-redundant defense/stress annotation groups. A total of 50 non-redundant defense gene IDs were found to be induced between the two datasets (S6A Table). Only eight of the 22 common gene IDs showed identical patterns of induction for the three *Pratylenchus* treatments on the two datasets. Fourteen gene IDs were unique to the coding sequence-based dataset and 13 were unique to the genome-based dataset. These “unique” gene IDs were usually present in the alternative ≥51 RPKM dataset, but the RPKM values did not meet the log2 fold-change criterion for induction. While the results indicated the potential importance of the mapping database used to assign gene IDs and annotations, they also indicated that strongly-induced defense/stress gene IDs were present in both datasets.

As a consequence of updating the coding sequence-based Traes gene ID numbers for this study, two different sets of gene annotations resulted, the original and one based on the wheat genome. Some of the original contig data (RPKM) assigned to certain gene IDs were shuffled, some were not present in the genome-based set, and novel gene IDs emerged. Also, annotations in the original set tended to be more detailed, such that there were more annotation groups representing the same general defense gene or gene family. Annotations for alternative splicing/mRNA isoforms could vary between the coding sequence and genome databases as well. Alternative splicing has been identified in *Triticum aestivium* in response to drought, heat and salt stress [43,44] and biotic stress [45]. This possibility would need to be verified using updated sequencing and manual curation [46,47]. Annotation disparities made it difficult to predict the qRT-PCR results based on priming sites in gene ID sequences. For instance, TraesCS1B01G249000 (lichenase) was absent in the genome-based set, and was instead found as a beta-1,3-glucanase in a primer binding site analysis. Of 360 non-redundant defense gene IDs in the combined sets, 158 (43%) were annotated with identical or nearly-identical descriptions. Each gene ID set harbored unique gene descriptions, indicating that two annotation sets were better than one.

### Selected root defense genes

**Fig 3:**
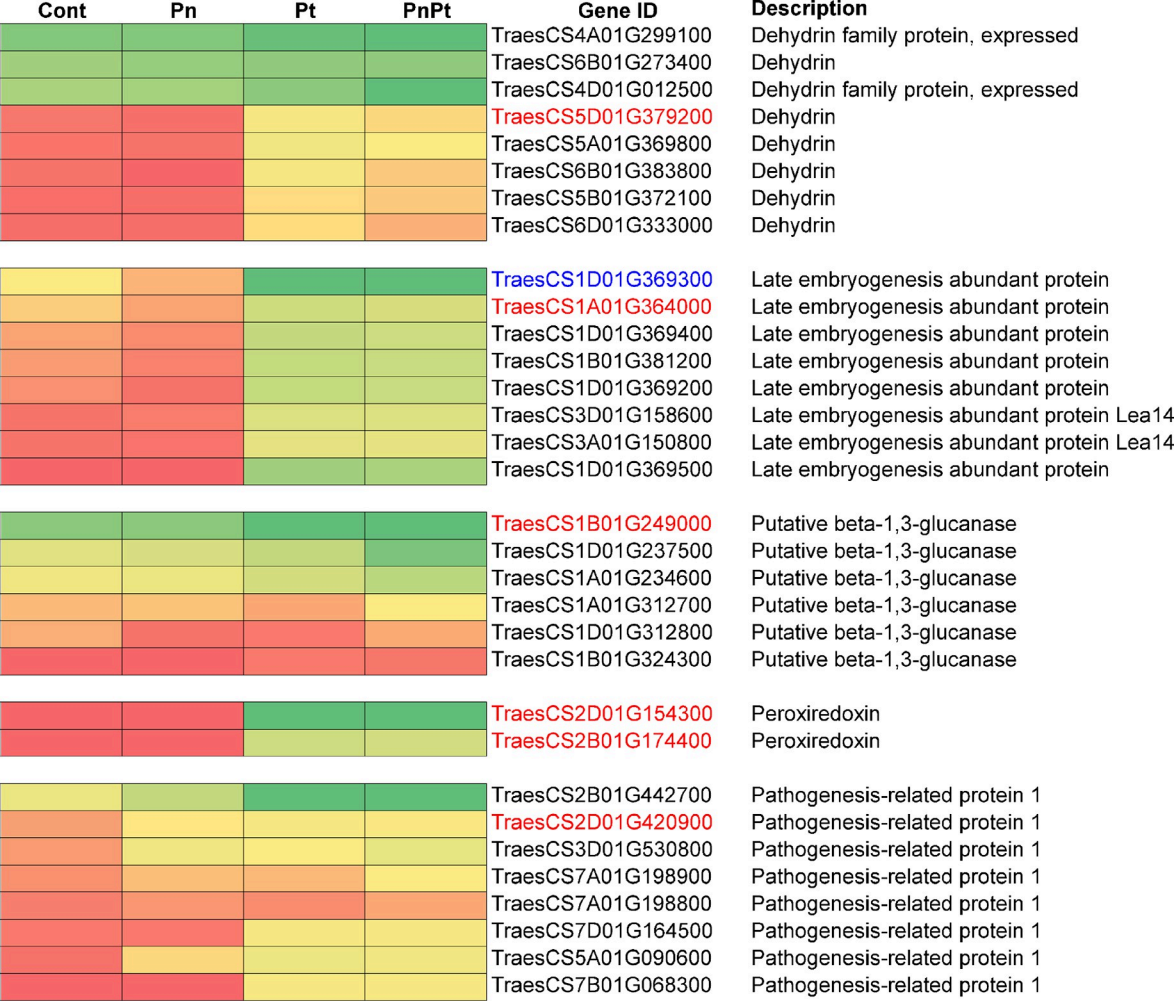
Heat maps of RPKM values for selected defense genes expressed in roots of wheat cultivar Scarlet 7 days after inoculation with *Pratylenchus neglectus* (Pn), *P*. *thornei* (Pt) and a mixture of both species (PnPt). The control treatment was water (Cont). Plots were derived from genome-based gene IDs having total RPKM values ≥51 (total = sum of RPKM values of all four treatments). High relative RPKM values are shown in shades of green, low values are indicated by orange or red, and yellow shades indicate intermediate values. Gene IDs in colored font indicate sequences used for primer design.

Proteins encoding Dehydrin2, Late embryogenesis-abundant proteins 76 and group 3, (known as -(1–3),(1–4)-β-D), Pathogenesis-related protein 1, and 1-cys-Peroxiredoxin represent several mechanisms of defense against abiotic and biotics stress in plants [25]. Expression patterns for family members of these six selected defense genes from the genome-based dataset are shown as heat maps (Fig 3). Members that were strongly expressed, induced or repressed in any of the *Pratylenchus* treatments were included; constitutively-expressed members showing moderate levels of expression were omitted. In the *Dehydrin* family, three members appeared to be strongly constitutively expressed, whereas four were induced upon Pt and Pn+Pt treatments but not with Pn. The Pn treatment resembled the control (water) in all families except Pathogenesis-related protein1. The data suggest that Pn was not substantially activating gene expression under the experimental conditions of this project.

*Pratylenchus*-treated and control roots of Scarlet and Rz1 were analyzed for expression of six defense genes in qRT-PCR assays. Primers used in this study, shown in Table 2, were designed for a subset of gene family members that previously displayed gene induction [31].

**Table 2:** qRT-PCR primers used in this study.

Target^a^	Primers	Sequence (5’ to 3’)	Annealing	Amplicon
			temp (°C)^b^	(bp)^c^
***Dehydrin***	DHN-F	TCCAGCTCCAGCTCGTCTGAGGA	66	220
	DHN-R	GCTTCTCCTTGATCTTGTCCATGAC		
***LEA 76***	LEAv1-F	GACAAAACCGGCAGCGTGCTCCAGCA	74	125
	LEAv1-R	GTGGTGGTGTCCTTGGTGGTGTTGTCAC		
***LEA grp3***	LEAv4-F	AAGACGGAGGCGGCCAAGCAGAA	72	269
	LEAv4-F	GTGGTGTTGTCCTTGGTGGTGTTGGTGTTG		
***Lichenase***	Lic-F	CAACGTGAAGCTCGTGGTGTCGGAG	68	214
	Lic-R	GTAGAAGAGTCCCCAGTTCTGCTCCA		
***Peroxiredoxin***	Per-F1	CCATCAAGCAACTGAACATGGTCG	68	233
	Per-F2	CCATCAAGCAGCTCAACATGGTCG		
	Per-R	GAACCCCTGCGGGAACATCTTCTT		
***PR-1 protein***	PR1-F	GTCATGGACTGGCACACGCT	66	159
	PR1-R	CTCCACCTTGATGCACGACGTG		
***TEF1***	EF1a-F	GATGATCTGCTGCTGCAACAAGATGGA	67	199
	EF1a-R	GCCCTTGTACCAGTCAAGGTTGG		

^a^ LEA, late embryogenesis-abundant protein; PR-1 protein, pathogenesis-related protein1/major pollen allergen family; TEF1, eukaryotic translation elongation factor EF-1 alpha (internal standard).

^b^ Determined using the online OligoAnalyzer^TM^ tool from Integrated DNA Technologies (Coralville, Iowa, United States of America).

^c^ Determined from the length of the cDNA between and inclusive of the primer binding sites.

**Fig 4:**
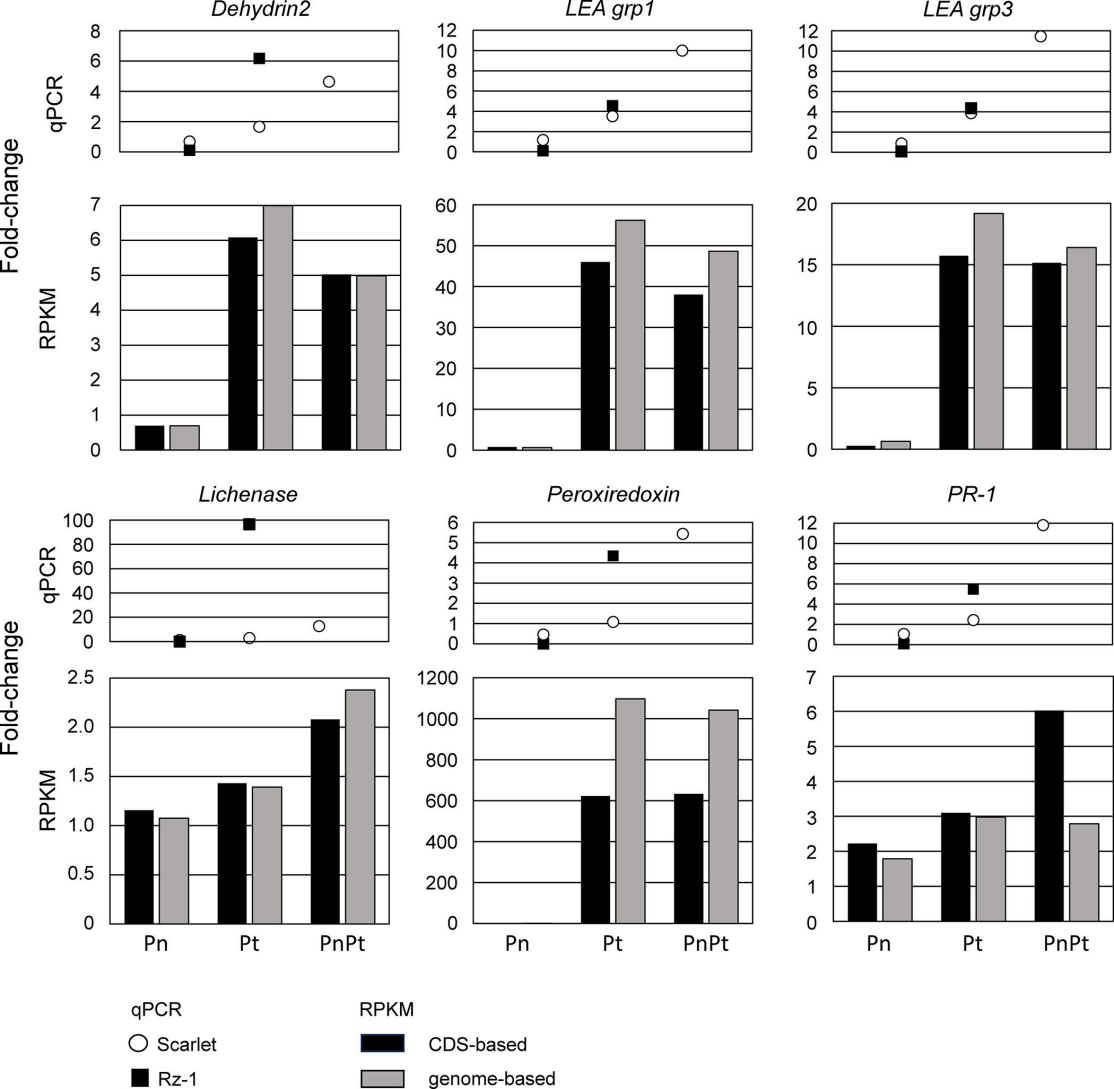
Comparison of defense gene induction in roots of Scarlet and Scarlet-Rz1 after treatment with *Pratylenchus neglectus* (Pn), *P*. *thornei* (Pt) or a combination of both species (PnPt). The qRT-PCR relative fold-change data (qPCR) are shown in the upper panels. Open circles represent Scarlet and the black squares represent Scarlet-Rz1. The Pn+Pt combination for Rz1 was omitted because its cDNA failed to amplify in any of the assays. Coding sequence-based and genome-based RPKM data (RPKM) are shown as black and grey bars, respectively, in the lower panels.

Roots of Scarlet and Rz1 inoculated with *Pratylenchus* spp. or treated with (control) were analyzed for expression of these defense genes in qRT-PCR assays. The qRT-PCR relative fold-change values for each gene and nematode treatment are as single icons (Fig 4, upper panels**)**. Fold-change values for each treatment, generated from coding sequence-based and genome-based RPKM values, were plotted as bar graphs (Fig 4, bottom panels). Data used to calculate all fold-change values are shown in S8 Table.

The qRT-PCR data indicated that transcripts were the most abundant in the Pn+Pt treatments and least abundant in the Pn treatment. Expression of *LEA76*, *LEA grp3*, *PR-1* and *peroxiredoxin* in Scarlet and Rz1 were similar, but for the Pt treatment, *Dehydrin2* and *Lichenase* were more highly expressed in Rz1 compared to Scarlet. The RPKM data showed that *P*. *thornei* induced gene expression to a greater degree than did *P*. *neglectus* in Scarlet. However, the qRT-PCR-based and RPKM-based fold-change assessments differed in the magnitude of the fold-change, and how the fold-change values compared among treatments. For instance, there were disparities among the qRT-PCR and RPKM assessments in the magnitude of the fold-change for the Pn treatment relative to PnPt. It is possible that the qRT-PCR assays were detecting additional gene IDs beyond those that were identified for primer design. Bar graphs derived from genome-based RPKM values showed similar trends to those derived from the coding sequence-based gene IDs, but the magnitude of the fold-change values of the former were greater than that of the latter in eight cases (Fig 4).

The qRT-PCR assays, which amplified one or two members of each gene family, authenticated transcriptome data for six defense-related genes not observed in *Pseudomonas-Triticum* root interactions and *Fusarium avenaceum-Avena fatua s*eed interactions. In the former, host genes encoding components of the jasmonate pathway, hypersensitive reaction and ROS detoxification were highly induced after colonization by a biocontrol bacterium [48,49]. In the latter, host genes involved in ROS detoxification, xenobiotic export and detoxification, and production of antifungal proteins and metabolites were induced in response to a fungal pathogen of seeds [50,51]. The uniqueness of induced genes in the various interactions indicates that plant defense depends on the organ under attack and the type of interacting microbe.

*Pratylenchus neglectus* and *P*. *thornei* are migratory endoparasites that move between soil and wheat roots, reducing root health, host viability and productivity. These nematodes intracellularly migrate through root cortical cells without disrupting the lignin and suberin layers of the vascular cylinder [52,53]. Under moderate levels of infestation, this mode of parasitism allows the host plant a margin of survival during which the nematode can reproduce. Despite common pathogenicity strategies, the *Pratylenchus* used in this study differed in several aspects. More *P*. *neglectus* was associated with roots of both Scarlet and fungal-resistant Rz-1 compared to *P*. *thornei* after a 7-day treatment. However, the *P*. *neglectus* treatment induced a fewer number of defense/stress genes relative to the *P*. *thornei* treatment (Table 1), and the magnitude of induction of selected defense genes generally was lower for *P*. *neglectus* compared to *P*. *thornei* (Fig 4). The data suggest that, under the conditions of this study, wheat roots mounted a bigger defense response to *P*. *thornei* that resulted in decreased nematode populations in the root. The genotype Rz-1 showed a greater fold-change in expression of *Dehydrin2*, *Lichenase* and *Peroxiredoxin* compared to Scarlet in the *Pt* treatment (Fig 4), but there were no obvious differences between the Scarlet and Rz-1 *Pratylenchus* densities (Fig 1). A rigorous determination of nematode resistance of both genotypes should be conducted before any correlations can be made between defense gene expression and *Pratylenchus* resistance. However, the data from Scarlet indicate that *P*. *neglectus* and *P*. *thornei* differed in infection dynamics (timing of defense gene expression in the roots) and might involve different defense programs (types of defense genes).

Innovations in DNA marker technology and development of new types of mapping populations are providing a more global picture of defense gene candidates and their locations in the wheat genome. Two potential defense genes induced in this study, *CYP450* and *C2H2 Zn finger*, were previously associated with resistance to *P*. *thornei* [14]. Wheat breeders might also benefit from studies in other crop plants. A gene encoding a Bidirectional sugar transporter SWEET1 induced by *P*. *thornei* in a resistant chickpea cultivar [54] was also found in this study. Finally, this study reveals candidate promoters, including those from *Dehydrin2*, *Late embryogenesis-abundant* group 3 and *1-cys-Peroxiredoxin*, for driving *Pratylenchus*-inducible defense gene expression in wheat roots, and several defense genes that are common to both *P*. *thornei* and *P*. *neglectus* responses.

## Conclusions

Our findings indicate that root *Pratylenchus* population density and induction of specific defense genes is specific to the nematode species. The findings provide a basis to explore the relationship between cultivar, nematode population densities, and host defense genes expression under a variety of environmental conditions, including the field. Meanwhile, prospects are positive with respect to novel genetic sources for control of *P*. *thornei* and *P*. *neglectus*. There are several defense genes common to both the *P*. *thornei* and *P*. *neglectus* responses that might be deployed for resistance against both species. Promoters from three of the genes are promising candidates for driving resistance gene expression in wheat.

## Supporting information

S1 Table Coding sequence-based gene IDs. (XLSX)

S2 Table Genome-based gene IDs. (XLSX)

S3 Table Coding sequence-based defense gene IDs. (XLSX)

S4 Table Genome-based defense gene IDs. (XLSX)

S5 Table References for defense gene IDs. (DOCX)

S6 Table qRT-PCR & RPKM fold-change data for Fig 4. (XLSX)

S7 Table Nematode enumeration data for Fig 1. (XLSX)

S8 Table Quantitative RT-PCR and RPKM fold-change data for Fig 4. (XLSX)
