# Supplementary material for: Differential induction of defense genes in hexaploid wheat roots by the plant-parasitic nematodes Pratylenchus neglectus and P. thornei
Source: PLoS One. 2024 Aug 29;19(8):e0306533. doi: 10.1371/journal.pone.0306533 (PMC11361681; doi:10.1371/journal.pone.0306533)
Supplement: S5 Table — (DOCX) [file pone.0306533.s005.docx]

**S5 Table. References for defense gene IDs in S3 & S4 Tables**

Please refer to the end of this file for multiple annotations that use the same reference under

**Disease resistance proteins**

**Pathogenesis-related proteins**

**Oxidative stress and reactive oxygen species (ROS)**

**Sterol biosynthesis**

**1-aminocyclopropane-1-carboxylate oxidase**

Jolien Pattyn, John Vaughan-Hirsch, Bram Van de Poel (2021) The regulation of ethylene biosynthesis: a complex multilevel control circuitry. New Phytol 229: 770–782. doi: 10.1111/nph.16873

**1-Deoxy-D-xylulose 5-phosphate reductoisomerase**

Proteau PJ (2004) 1-Deoxy-D-xylulose 5-phosphate reductoisomerase: an overview. Bioorg Chem 32(6): 483-493. doi: 10.1016/j.bioorg.2004.08.004

**1-Deoxy-D-xylulose 5-phosphate synthase 1**

Lichtenthaler HK (1999) The 1-deoxy-d-xylulose-5-phosphate pathway of isoprenoid biosynthesis in plants. Annu Rev Plant Physiol Plant Mol Biol 50: 47–65.

**2-C-methyl-D-erythritol 2,4-cyclodiphosphate synthase**

González-Cabanelas D, Wright LP, Paetz C, Onkokesung N, Gershenzon J, Rodríguez-Concepción M, Phillips MA (2015) The diversion of 2-C-methyl-D-erythritol-2,4-cyclodiphosphate from the 2-C-methyl-D-erythritol 4-phosphate pathway to hemiterpene glycosides mediates stress responses in *Arabidopsis thaliana*. Plant J 82(1): 122-137. doi: 10.1111/tpj.12798

Nawaporn Onkokesung, Michael Reichelt, Louwrance P Wright, Michael A Phillips, Jonathan Gershenzon, Marcel Dicke (2019) The plastidial metabolite 2‐C‐methyl‐D‐erythritol‐2,4‐cyclodiphosphate modulates defence responses against aphids. Plant Cell Environ. 42: 2309–2323. DOI: 10.1111/pce.13538

**3-hydroxy-3-methylglutaryl coenzyme A reductase**

Cecilia Brunetti, Lucia Guidi, Federico Sebastiani, Massimiliano Tattini (2015) Isoprenoids and phenylpropanoids are key components of the antioxidant defense system of plants facing severe excess light stress. Envir Exp Bot 119: 54-62. <https://doi.org/10.1016/j.envexpbot.2015.04.007>

Jon A Friesen, Victor W Rodwell (2004) The 3-hydroxy-3-methylglutaryl coenzyme-A (HMG-CoA) reductases. Genome Biol 5: 248. http://genomebiology.com/2004/5/11/248

**3-hydroxyisobutyryl-CoA hydrolase-like protein 1, mitochondrial**

Target of thioredoxins in plant (Yoshida et al.); redox responsive protein (Balmant et al.); role in Mt redox sensing

Keisuke Yoshida, Ko Noguchi, Ken Motohashi, Toru Hisabori (2013) Systematic exploration of thioredoxin target proteins in plant mitochondria. Plant Cell Physiol. 54(6): 875–892. doi:10.1093/pcp/pct037

Kelly Mayrink Balmant, Jennifer Parker, Mi-Jeong Yoo, Ning Zhu, Craig Dufresne, Sixue Chen (2015) Redox proteomics of tomato in response *to Pseudomonas syringae* infection. Hort Res 2: 15043. doi:10.1038/hortres.2015.43

**3'-N-debenzoyl-2'-deoxytaxol N-benzoyltransferase**

# [Da Cheng Hao](https://link.springer.com/article/10.1007/s10709-008-9257-7#auth-Da_Cheng-Hao), [Ling Yang](https://link.springer.com/article/10.1007/s10709-008-9257-7#auth-Ling-Yang), [Beili Huang](https://link.springer.com/article/10.1007/s10709-008-9257-7#auth-Beili-Huang) (2009) Molecular evolution of paclitaxel biosynthetic genes *TS* and *DBAT* of *Taxus* species. [Genetica](https://link.springer.com/journal/10709) 135: 123–135.

Robert M Long, Chandraiah Lagisetti, Robert M Coates, Rodney B Croteau (2008) Specificity of the N-benzoyl transferase responsible for the last step of taxol biosynthesis. Arch Biochem Biophys 477(2): 384–389. doi:10.1016/j.abb.2008.06.021

**4-coumarate:CoA ligase**

**Cinnamoyl CoA reductase**

**Phenylalanine ammonia-lyase**

**Trans-cinnamate 4-monooxygenase**

Vivek Yadav, Zhongyuan Wang, Chunhua Wei, Aduragbemi Amo, Bilal Ahmed, Xiaozhen Yang, Xian Zhang (2020) Phenylpropanoid pathway engineering: an emerging approach towards plant defense. Pathogens 9: 312. doi:10.3390/pathogens9040312

**4-diphosphocytidyl-2-C-methyl-D-erythritol kinase**

Takashi Wada, Tomohisa Kuzuyama, Shinya Satoh, Seiki Kuramitsu, Shigeyuki Yokoyama, Satoru Unzai, Jeremy RH Tame, Sam-Yong Park (2003) Crystal structure of 4-(cytidine 5’-diphospho)-2-*C*-methyl-D-erythritol kinase, an enzyme in the non-mevalonate pathway of isoprenoid synthesis. J Biol Chem 278(32): 30022–30027.

**4-hydroxy-3-methylbut-2-en-1-yl diphosphate synthase**

Ma D, Li G, Zhu Y and Xie D-Y (2017) Overexpression and suppression of *Artemisia annua* 4-Hydroxy- 3-Methylbut-2-enyl Diphosphate Reductase 1 gene (AaHDR1) differentially regulate artemisinin and terpenoid biosynthesis. Front. Plant Sci 8:77. doi: 10.3389/fpls.2017.00077

**4,5-DOPA dioxygenase extradiol**

Ge Li, Xiaoqing Meng, Mingku Zhu, Zongyun Li (2019) Research progress of betalain in response to adverse stresses and evolutionary relationship compared with anthocyanin. Molecules 24: 3078. doi:10.3390/molecules24173078

**10-deacetylbaccatin III 10-O-acetyltransferase**

# [Da Cheng Hao](https://link.springer.com/article/10.1007/s10709-008-9257-7#auth-Da_Cheng-Hao), [Ling Yang](https://link.springer.com/article/10.1007/s10709-008-9257-7#auth-Ling-Yang), [Beili Huang](https://link.springer.com/article/10.1007/s10709-008-9257-7#auth-Beili-Huang) (2009) Molecular evolution of paclitaxel biosynthetic genes *TS* and *DBAT* of *Taxus* species. [Genetica](https://link.springer.com/journal/10709) 135: 123–135.

**10 kDa chaperonin**

Akanksha Pareek, Divya Mishra, Divya Rathi, Jitendra Kumar Verma, Subhra Chakraborty, Niranjan Chakraborty (2021) The small heat shock proteins, chaperonin 10, in plants: an evolutionary view and emerging functional diversity. Environ Exp Bot 182: 104323. <https://doi.org/10.1016/j.envexpbot.2020.104323>

**12-oxophytodienoate reductase**

Jingjun Ruan, Yuexia Zhou, Meiliang Zhou, Jun Yan, Muhammad Khurshid, WenfengWeng, Jianping Cheng, Kaixuan Zhang (2019) Jasmonic acid signaling pathway in plants. Int J Mol Sci 20: 2479. doi:10.3390/ijms20102479

**14 kDa proline-rich protein DC2.15**

Duk Jun Yu, Sung Hoon Jun, Junhyung Park, Jung Hyun Kwon, Hee Jae Lee (2020) Transcriptome analysis of genes involved in cold hardiness of peach tree (*prunus persica*) shoots during cold acclimation and deacclimation. Genes 11: 611. doi:10.3390/genes11060611

and terpenoid biosynthesis. Front Plant Sci 8: 77. doi: 10.3389/fpls.2017.00077

**60 kDa chaperonin (HSP60)**

M Nagaraju, Anuj Kumar, N Jalaja, D Manohar Rao, PB Kavi Kishor (2021) Functional Exploration of chaperonin (HSP60/10) family genes and their abiotic stress-induced expression patterns in *Sorghum bicolor.* Curr Genom 22: 137-152. DOI:10.2174/1389202922666210324154336

**60S ribosomal proteins L12, L14, L19**

**60S ribosomal proteins L12 & L14**

**(Plant) ribosomal proteins RPL12 & RPL19**

**50S ribosomal protein L19**

**Ribosomal protein L19**

Nagaraj S, Senthil-Kumar M, Ramu VS, Wang K and Mysore KS (2016) Plant ribosomal proteins, RPL12 and RPL19, play a role in nonhost disease resistance against bacterial pathogens. Front Plant Sci 6: 1192. doi: 10.3389/fpls.2015.01192

**ABC transporters**

**ATP binding cassette proteins**

# [Anil Dahuja](https://onlinelibrary.wiley.com/authored-by/Dahuja/Anil), [Ranjeet R Kumar](https://onlinelibrary.wiley.com/authored-by/Kumar/Ranjeet+R.), [Akshay Sakhare](https://onlinelibrary.wiley.com/authored-by/Sakhare/Akshay), [Archana Watts](https://onlinelibrary.wiley.com/authored-by/Watts/Archana), [Bhupinder Singh](https://onlinelibrary.wiley.com/authored-by/Singh/Bhupinder), [Suneha Goswami](https://onlinelibrary.wiley.com/authored-by/Goswami/Suneha), [Archana Sachdev](https://onlinelibrary.wiley.com/authored-by/Sachdev/Archana), [Shelly Praveen](https://onlinelibrary.wiley.com/authored-by/Praveen/Shelly) (2021) Role of ATP-binding cassette transporters in maintaining plant homeostasis under abiotic and biotic stresses. Physiol Plantar 171(4): 785-801. <https://doi.org/10.1111/ppl.13302>

Thomas S Lane, Caroline S Rempe, Jack Davitt, Margaret E Staton, Yanhui Peng, Douglas Edward Soltis, Michael Melkonian, Michael Deyholos, James H Leebens-Mack, Mark Chase, Carl J Rothfels, Dennis Stevenson, Sean W Graham, Jun Yu, Tao Liu, J. Chris Pires, Patrick P Edger, Yong Zhang, Yinlong Xie, Ying Zhu, Eric Carpenter, Gane Ka-Shu Wong, C Neal Stewart Jr (2016) Diversity of ABC transporter genes across the plant kingdom and their potential utility in biotechnology. BMC Biotechnol 16: 47. DOI 10.1186/s12896-016-0277-6

**B family ABC transporters**

Flanagan JU, Huber T (2007) Structural evolution of the ABC transporter subfamily B. Evol Bioinform 3: 309-316.

**G family ABC transporters**

Gräfe K, Schmitt L (2021) The ABC transporter G subfamily in *Arabidopsis thaliana*. J Exp Bot 72(1): 92-106. doi: 10.1093/jxb/eraa260

Hao Ji, Yanhui Peng, Nicole Meckes, Sara Allen, C. Neal Stewart Jr, M. Brian Traw (2014) ATP-dependent binding cassette transporter G family member 16 increases plant tolerance to abscisic acid and assists in basal resistance against *Pseudomonas syringae* DC3000. Plant Physiol 166: 879–888. www.plantphysiol.org

**ABRE binding factor 4**

Pan W, Zheng P, Zhang C, Wang W, Li Y, Fan T, Liu Y, Cao S (2020) The effect of ABRE BINDING FACTOR 4-mediated FYVE1 on salt stress tolerance in Arabidopsis. Plant Sci 296: 110489. doi: 10.1016/j.plantsci.2020.110489

**Abscisic acid-insensitive5**

Collin A, Daszkowska-Golec A, Kurowska M and Szarejko I (2020) Barley ABI5 (Abscisic Acid INSENSITIVE 5) is involved in abscisic acid-dependent drought response. Front. Plant Sci 11: 1138. doi: 10.3389/fpls.2020.01138

Skubacz A, Daszkowska-Golec A and Szarejko I (2016) The role and regulation of ABI5 (ABA-Insensitive 5) in plant development, abiotic stress responses and phytohormone crosstalk. Front. Plant Sci 7: 1884. doi: 10.3389/fpls.2016.01884

**Abscisic acid receptor**

Nakashima K, Yamaguchi-Shinozaki K (2013) ABA signaling in stress-response and seed development. Plant Cell Rep 32: 959–970. https://doi.org/10.1007/s00299-013-1418-1

**Abscisic stress ripening**

Ting Zan, Liqun Li, Tingting Xie, Li Zhang, Xuejun Li (2020) Genome-wide identification and abiotic stress response patterns of abscisic acid stress ripening protein family members in *Triticum aestivum* L. Genomics 112: 3794–3802. https://doi.org/10.1016/j.ygeno.2020.04.007

Tuteja N (2007) Abscisic acid and abiotic stress signaling. Plant Signal Behav 2(3): 135-138. http://www.landesbioscience.com/journals/psb/abstract.php?id=4156

**Accelerated cell death 11**

Brodersen P, Petersen M, Pike HM, Olszak B, Skov S, Odum N, Jørgensen LB, Brown RE, Mundy J (2002) Knockout of Arabidopsis accelerated-cell-death11 encoding a sphingosine transfer protein causes activation of programmed cell death and defense. Genes Dev 16(4): 490-502. doi: 10.1101/gad.218202

**Acclimation protein (provisional)**

Viswanathan Chinnusamy, Jian-Kang Zhu, Ramanjulu Sunkar (2010) Gene regulation during cold stress acclimation in plants. Methods Mol Biol 639: 39–55. doi:10.1007/978-1-60761-702-0_3

**Acid invertase**

Ning Jiang, Pinghui Yu, Weimeng Fu, Guangyan Li, Baohua Feng, Tingting Chen, Hubo Li, Longxing Tao, Guanfu Fu (2020) Acid invertase confers heat tolerance in rice plants by maintaining energy homoeostasis of spikelets. Plant Cell Environ. 43: 1273–1287. DOI: 10.1111/pce.13733

**Activator of 90 kDa heat shock ATPase-like protein**

Saeed ul Haq, Abid Khan, Muhammad Ali, Abdul Mateen Khattak, Wen-Xian Gai, Huai-Xia Zhang, Ai-Min Wei, Zhen-Hui Gong (2019). Heat shock proteins: dynamic biomolecules to counter plant biotic and abiotic stresses. Int J Mol Sci 20: 5321. doi:10.3390/ijms20215321

**Agmatine deiminase**

Janowitz T, Kneifel H, Piotrowski M (2003) Identification and characterization of plant agmatine iminohydrolase, the last missing link in polyamine biosynthesis of plants. FEBS Lett 544(1-3): 258-261. doi: 10.1016/s0014-5793(03)00515-5

Liu C, Atanasov KE, Arafaty N, Murillo E, Tiburcio AF, Zeier J, Alcázar R (2020) Putrescine elicits ROS-dependent activation of the salicylic acid pathway in *Arabidopsis thaliana*. Plant Cell Environ 43(11): 2755-2768. doi: 10.1111/pce.13874

**AIG2-like protein**

Zhixue Wang, [Leiyun Yang](javascript:;), [Georg Jander](javascript:;), [Ruchika Bhawal](javascript:;), [Sheng Zhang](javascript:;), [Zhenhua Liu](javascript:;), [Aaron Oakley](javascript:;), [Jian Hua](javascript:;) (2022) AIG2A and AIG2B limit the activation of salicylic acid-regulated defenses by tryptophan-derived secondary metabolism in Arabidopsis, Plant Cell 34(11): 4641–4660. [https://doi.org/10.1093/plcell/koac255](https://doi.org/10.1093/plcell/koac255v)

**Alanine:glyoxylate aminotransferase**

# [Eunsook Chung](https://link.springer.com/article/10.1007/BF03191133#auth-Eunsook-Chung), [Kyoung Mi Kim](https://link.springer.com/article/10.1007/BF03191133#auth-Kyoung_Mi-Kim), [Jee Eun Heo](https://link.springer.com/article/10.1007/BF03191133#auth-Jee_Eun-Heo), [Chang-Woo Cho](https://link.springer.com/article/10.1007/BF03191133#auth-Chang_Woo-Cho), [Seon-Woo Lee](https://link.springer.com/article/10.1007/BF03191133#auth-Seon_Woo-Lee), [Jai-Heon Lee](https://link.springer.com/article/10.1007/BF03191133#auth-Jai_Heon-Lee) (2009) Molecular characterization of mungbean peroxisomal alanine glyoxylate aminotransferase gene induced by low temperature stress. [Genes Genom](https://link.springer.com/journal/13258) 31: 11–18.

**Aldehyde dehydrogenase**

Tola AJ, Jaballi A, Germain H, Missihoun TD (2021) Recent development on plant aldehyde dehydrogenase enzymes and their functions in plant development and stress signaling. Genes 12: 51. <https://doi.org/10.3390/genes12010051>

**Aldehyde oxidase**

Wu J, Kamanga BM, Zhang W, Xu Y, Xu L (2022) Research progress of aldehyde oxidases in plants. PeerJ 10: e13119. DOI 10.7717/peerj.13119

**Aldo-keto reductase superfamily**

**Aldo/keto reductase family oxidoreductase**

Oleg A Barski, Srinivas M Tipparaju, Aruni Bhatnagar (2008) the aldo-keto reductase superfamily and its role in drug metabolism and detoxification. Drug Metab Rev 40(4): 553–624. doi:10.1080/03602530802431439

**Alkaline ceramidase**

Li-Qun Huang, Ping-Ping Li, Jian Yin, Yong-Kang Li, Ding-Kang Chen, He-Nan Bao, Rui-Yuan Fan, Hao-Zhuo Liu, Nan Yao (2022) Arabidopsis alkaline ceramidase ACER functions in defense against insect herbivory. J Exp Bot 73(14): 4954–4967. https://doi.org/10.1093/jxb/erac166

Jian Li, Jian Yin, Jian-Xin Wu, Ling-Yan Wang, Yu Liu, Li-Qun Huang, Rui-Hua Wang, Nan Yao (2022) Ceramides regulate defense response by binding to RbohD in Arabidopsis. Plant J 109: 1427–1440. doi: 10.1111/tpj.15639

**Alkyl hydroperoxide reductase subunit C**

Zhang B, Gu H, Yang Y, Bai H, Zhao C, Si M, Su T, Shen X (2019) Molecular mechanisms

of AhpC in resistance to oxidative stress in *Burkholderia thailandensis*. Front Microbiol 10: 1483. doi: 10.3389/fmicb.2019.01483

**Allene oxide cyclase**

**Allene oxide synthase**

Jingjun Ruan, Yuexia Zhou, Meiliang Zhou, Jun Yan, Muhammad Khurshid, WenfengWeng, Jianping Cheng, Kaixuan Zhang (2019) Jasmonic acid signaling pathway in plants. Int J Mol Sci 20: 2479. doi:10.3390/ijms20102479

**Alliin lyase/Allicin**

JC Harris, SL Cottrell, S Plummer, D Lloyd (2001) Antimicrobial properties of *Allium sativum* (garlic). Microbiol Biotechnol 57:282–286. DOI 10.1007/s002530100722

**Altered inheritance of mitochondria protein (Aim32)**

Danyun Zhang, Owen R Dailey, Daniel J Simon, Kamilah Roca-Datzer, Yasaman Jami-Alahmadi, Mikayla S Hennen, James A Wohlschlegel, Carla M Koehler, Deepa V Dabir (2021) Aim32 is a dual-localized 2Fe-2S mitochondrial protein that functions in redox quality control. J Biol Chem 297(4): 101135. https://doi.org/10.1016/j.jbc.2021.101135

**Aluminium induced protein with YGL and LRDR motifs** **(AILP1)**

No publication available

**Aluminum sensitive 3**

Larsen PB, Geisler MJ, Jones CA, Williams KM, Cancel JD (2005) ALS3 encodes a phloem-localized ABC transporter-like protein that is required for aluminum tolerance in Arabidopsis. Plant J 41(3): 353-363. doi: 10.1111/j.1365-313X.2004.02306.x

**Amine oxidase**

Alessandra Cona, Giuseppina Rea, Riccardo Angelini, Rodolfo Federico, Paraskevi Tavladoraki (2006) Functions of amine oxidases in plant development and defence. Trends Plant Sci 11(2): 80-88. doi:10.1016/j.tplants.2005.12.009

**Ankryin-repeat protein**

Zhao J-Y, Lu Z-W, Sun Y, Fang Z-W, Chen J, Zhou Y-B, Chen M, Ma Y-Z, Xu Z-S and Min D-H (2020) The ankyrin-repeat gene GmANK114 confers drought and salt tolerance in Arabidopsis and soybean. Front. Plant Sci 11: 584167. doi: 10.3389/fpls.2020.584167

**AP2-like ethylene-responsive transcription factor**

Phukan UJ, Jeena GS, Tripathi V, Shukla RK (2017) Regulation of Apetala2/Ethylene Response Factors in Plants. Front Plant Sc. 8: 150. doi: 10.3389/fpls.2017.00150.

Xie Z, Nolan TM, Jiang H, Yin Y (2019) AP2/ERF transcription factor regulatory networks in hormone and abiotic stress responses in *Arabidopsis*. Front Plant Sci 10: 228. doi: 10.3389/fpls.2019.00228

**Apoptosis inhibitor 5**

Erick J Morris, William A Michaud, Jun-Yuan Ji, Nam-Sung Moon, James W Rocco, Nicholas J Dyson (2006) Functional identification of Api5 as a suppressor of E2F-dependent apoptosis in vivo. PloS Genet 2(11): e196. doi:10.1371/journal.pgen.0020196

**Apoptosis-inducing factor**

Eric Daugas, Dominique Nochy, Luigi Ravagnan, Markus Loeffer, Santos A Susina, Naoufal Zamzami. Guido Kroemer (2000) Apoptosis-inducing factor (AIF): a ubiquitous mitochondrial oxidoreductase involved in apoptosis. FEBS Lett 476: 118-123.

# Aquaporin

# Zunaira Afzal, TC Howton, Yali Sun, [M. Shahid Mukhtar](https://sciprofiles.com/profile/2215104) (2016) The roles of aquaporins in plant stress responses. J Dev Biol 4(1): 9. <https://doi.org/10.3390/jdb4010009>

**ARF GAP-like zinc finger-containing protein ZIGA4**

Yu J, Gonzalez JM, Dong Z, Shan Q, Tan B, Koh J, Zhang T, Zhu N, Dufresne C, Martin GB, Chen S (2021) Integrative proteomic and phosphoproteomic analyses of pattern- and effector-triggered immunity in tomato. Front Plant Sci 12: 768693. doi: 10.3389/fpls.2021.768693

**Arginine decarboxylase**

Rossi FR, Marina M, Pieckenstain FL (2015) Role of Arginine decarboxylase (ADC) in *Arabidopsis thaliana* defence against the pathogenic bacterium *Pseudomonas viridiflava*. Plant Biol (Stuttg). 17(4): 831-839. doi: 10.1111/plb.12289.

**Arsenate reductase**

Chao D-Y, Chen Y, Chen J, Shi S, Chen Z, Wang C, Danku JM, Zhao F-J, Salt DE (2014) Genome-wide association mapping identifies a new arsenate reductase enzyme critical for limiting arsenic accumulation in plants. PLoS Biol 12(12): e1002009. doi:10.1371/journal.pbio.1002009

**Arsenite transport protein**

Ali W, Isayenkov SV, Zhao FJ, Maathuis FJ (2009) Arsenite transport in plants. Cell Mol Life Sci 66(14): 2329-2339. doi: 10.1007/s00018-009-0021-7.

**AtMIN7**

**Brefeldin A-inhibited guanine nucleotide-exchange protein 5**

Zhao Z, Yang X, Lü S, Fan J, Opiyo S, Yang P, Mangold J, Mackey D, Xia Y (2022) Deciphering the novel role of AtMIN7 in cuticle formation and defense against the bacterial pathogen infection. Int J Mol Sci 21(15): 5547. doi: 10.3390/ijms21155547

Kinya Nomura, Christy Mecey, Young-Nam Lee, Lori Alice Imboden, Jeff H Chang, Sheng Yang Hea (2011) Effector-triggered immunity blocks pathogen degradation of an immunity-associated vesicle traffic regulator in *Arabidopsis*. Proc Natl Acad Sci USA 108(26): 10774–10779. www.pnas.org/cgi/doi/10.1073/pnas.1103338108

**Autophagy 8E**

Yan Y, Wang P, He C, Shi H (2017) MeWRKY20 and its interacting and activating autophagy-related protein 8 (MeATG8) regulate plant disease resistance in cassava. [Communications](https://www.sciencedirect.com/journal/biochemical-and-biophysical-research-communications) 4494(1-2): 20-26. <https://doi.org/10.1016/j.bbrc.2017.10.091>

**Autophagy-related protein 13**

Su T, Li X, Yang M, Shao Q, Zhao Y, Ma C, Wang P (2020) Autophagy: an intracellular degradation pathway regulating plant survival and stress response. Front Plant Sci 11: 164.

doi: 10.3389/fpls.2020.00164

**Auxilin-related protein 1**

Chang-Jin Park, Tong Wei, Rita Sharma, Pamela C Ronald (2017) Overexpression of rice auxilin-like protein, xb21, induces necrotic lesions, up-regulates endocytosis-related genes, and confers enhanced resistance to *Xanthomonas oryzae* pv. *oryzae*. Rice 10: 27. DOI 10.1186/s12284-017-0166-1

**Avr9/Cf-9 rapidly elicited protein**

Rowland O, Ludwig AA, Merrick CJ, Baillieul F, Tracy FE, Durrant WE, Fritz-Laylin L, Nekrasov V, Sjölander K, Yoshioka H, Jones JD (2005) Functional analysis of Avr9/Cf-9 rapidly elicited genes identifies a protein kinase, ACIK1, that is essential for full Cf-9-dependent disease resistance in tomato. Plant Cell 17(1): 295-310. doi: 10.1105/tpc.104.026013

**B12D protein**

Almutairi ZB (2022) In silico identification and characterization of B12D family proteins in Viridiplantae. Evol Bioinform 18: 1–12. DOI: 10.1177/11769343221106795

**Baculoviral IAP repeat-containing protein 3**

# Juan Tian, Xueyan Zhang, Benguo Liang, Shanwei Li, Zhixia Wu, Qianhua Wang, Chunxu Leng, Jiangli Dong (2010) Expression of baculovirus anti-apoptotic genes *p35* and *op-iap* in cotton (*Gossypium hirsutum* L.) enhances tolerance to Verticillium wilt. PLoS ONE 5(12): e14218. doi:10.1371/journal.pone.0014218

**BAG (Bcl-associated athanogene) chaperonin**

**BAG family molecular chaperone regulator**

Arif M, Li Z, Luo Q, Li L, Shen Y, Men S (2021). The BAG2 and BAG6 genes are involved in multiple abiotic stress tolerances in *Arabidopsis thaliana*. Int J Mol Sci 22: 5856. https://doi.org/ 10.3390/ijms22115856

**BAX inhibitor1**

T Ishikawa, N Watanabe, M Nagano1, M Kawai-Yamada, E Lam (2011) Bax inhibitor-1: a highly conserved endoplasmic reticulum-resident cell death suppressor. Cell Death Differ 18: 1271–1278. doi:10.1038/cdd.2011.59

**Benzyl alcohol O-benzoyltransferase**

Widhalm JR, Dudareva N (2015) A familiar ring to it: biosynthesis of plant benzoic acids. Mol. Plant 8: 83–97. http://dx.doi.org/10.1016/j.molp.2014.12.001

**beta-D-xylosidase 4**

**Beta-xylosidase**

Bauer K, Nayem S, Lehmann M, Wenig M, Shu L-J, Ranf S, Geigenberger P, Vlot AC (2023) b-D-XYLOSIDASE 4 modulates systemic immune signaling in *Arabidopsis thaliana*. Front Plant Sci 13: 1096800. doi: 10.3389/fpls.2022.1096800

**beta-Lactamase**

Tooke CL, Hinchliffe P, Bragginton EC, Colenso CK, Hirvonen VHA, Takebayashi Y, Spencer J (2019) β-Lactamases and β-lactamase inhibitors in the 21st century. J Mol Biol 431(18): 3472-3500. doi: 10.1016/j.jmb.2019.04.002

# Betaine aldehyde dehydrogenase

Manabu Ishitani, Toshihide Nakamura, Seung Youn Hart, Tetsuko Takabe (1995) Expression of the betaine aldehyde dehydrogenase gene in barley in response to osmotic stress and abscisic acid. Plant Mol Biol 27: 307-315.

Elizabeth A Weretilnyk, Andrew D Hanson (1990) Molecular cloning of a plant betaine-aldehyde dehydrogenase, an enzyme implicated in adaptation to salinity and drought. Proc Natl Acad Sci USA 87: 2745-2749.

**Bidirectional sugar transporter SWEET**

Breia R, Conde A, Badim H, Fortes AM, Gerós H, Granell A (2021) Plant SWEETs: from sugar transport to plant-pathogen interaction and more unexpected physiological roles. Plant Physiol 186(2): 836-852. doi: 10.1093/plphys/kiab127.

**Bifunctional inhibitor/lipid-transfer protein/seed storage 2S albumin superfamily protein**

Marc J Champigny, Marisa Isaacs, Philip Carella, Jennifer Faubert, Pierre R Fobert, Robin K Cameron (2013) Long distance movement of DIR1 and investigation of the role of DIR1-like during systemic acquired resistance in *Arabidopsis*. Front Plant Sci 4: 230. doi: 10.3389/fpls.2013.00230

Marc J Champigny, Heather Shearer, Asif Mohammad, Karen Haines, Melody Neumann, Roger Thilmony, Sheng Yang He, Pierre Fobert, Nancy Dengler, Robin K Cameron (2011) Localization of DIR1 at the tissue, cellular and subcellular levels during Systemic Acquired Resistance in *Arabidopsis* using DIR1:GUS and DIR1:EGFP reporters. BMC Plant Biol 11: 125. <http://www.biomedcentral.com/1471-2229/11/125>

**Bifunctional pinoresinol-lariciresinol reductase**

# [Hannaneh Tashackori](https://pubmed.ncbi.nlm.nih.gov/?term=Tashackori+H&cauthor_id=30537598), [Mohsen Sharifi](https://pubmed.ncbi.nlm.nih.gov/?term=Sharifi+M&cauthor_id=30537598), [Najmeh Ahmadian Chashmi](https://pubmed.ncbi.nlm.nih.gov/?term=Ahmadian+Chashmi+N&cauthor_id=30537598), [Elisabeth Fuss](https://pubmed.ncbi.nlm.nih.gov/?term=Fuss+E&cauthor_id=30537598), [Mehrdad Behmanesh](https://pubmed.ncbi.nlm.nih.gov/?term=Behmanesh+M&cauthor_id=30537598), [Naser Safaie](https://pubmed.ncbi.nlm.nih.gov/?term=Safaie+N&cauthor_id=30537598) (2019) RNAi-mediated silencing of pinoresinol lariciresinol reductase in Linum album hairy roots alters the phenolic accumulation in response to fungal elicitor, J Plant Physiol 232: 115-126. doi: 10.1016/j.jplph.2018.11.005

**Bifunctional polymyxin resistance protein ArnA**

Sonawane KD, Parulekar RS, Malkar RS, Nimbalkar PR, Barage SH, Jadhav DB (2015) Homology modeling and molecular docking studies of ArnA protein from *Erwinia amylovora*: role in polymyxin antibiotic resistance. J. Plant Biochem Biotechnol 24: 425–432. https://doi.org/10.1007/s13562-014-0293-3

**BolA-like family protein**

Lu Qin, Meihuan Wang, Jia Zuo, Xiangyang Feng, Xuejiao Liang, Zhigeng Wu (2015) Cytosolic BolA plays a repressive role in the tolerance against excess iron and MV-induced oxidative stress in plants. PloS ONE 10(4): e0124887. doi:10.1371/journal.pone.0124887

**BRCA1-A**

Oliver Trapp, Katharina Seeliger, Holger Puchta (2011) Homologs of breast cancer genes in plants. Front Plant Sci 2: 9. doi: 10.3389/fpls.2011.00019

**Cadmium resistance 2**

Lin J, Gao X, Zhao J, Zhang J, Chen S and Lu L (2020) Plant Cadmium Resistance 2 (SaPCR2) facilitates cadmium efflux in the roots of hyperaccumulator *Sedum alfredii* Hance. Front Plant Sci 11: 568887. doi: 10.3389/fpls.2020.568887

**Cadmium tolerant 1**

Cai Z, Xian P, Wang H, Lin R, Lian T, Cheng Y, Ma Q, Nian H (2020) Transcription factor GmWRKY142 confers cadmium resistance by up-regulating the Cadmium Tolerance 1-like genes. Front Plant Sci 11: 724. doi: 10.3389/fpls.2020.00724

**Caffeoyl-CoA 3-O-methyltransferase**

Minxia Wang, Xiuliang Zhu, Ke Wang, Chungui Lu, Meiying Luo, Tianlei Shan, Zengyan Zhang (2018) A wheat caffeic acid 3-O-methyltransferase TaCOMT-3D positively contributes to both resistance to sharp eyespot disease and stem mechanical strength. Sci Rep 8: 6543. DOI:10.1038/s41598-018-24884-0

**Calcineurin B-like protein**

Kurusu T, Hamada J, Nokajima H, Kitagawa Y, Kiyoduka M, Takahashi A, et al. (2010a) Regulation of microbe-associated molecular pattern-induced hypersensitive cell death, phytoalexin production and defense gene expression by calcineurin B-like protein-interacting protein kinases, OsCIPK14/15, in rice cultured cells. Plant Physiol 153: 678–692. DOI: 10.1104/pp.109.151852

Addendum:

Takamitsu Kurusu, Jumpei Hamada, Haruyasu Hamada, Shigeru Hanamata, Kazuyuki Kuchitsu (2010b) Roles of calcineurin B-like protein-interacting protein kinases in innate immunity in rice

Plant Signal Behav 5(8): 1045-1047.

**Calcium-dependent lipid-binding (CaLB domain) family protein**

de Silva K, Laska B, Brown C, Sederoff HW, Khodakovskaya M (2011) Arabidopsis thaliana calcium-dependent lipid-binding protein (AtCLB): a novel repressor of abiotic stress response. J Exp Bot 62(8): 2679-2689. doi: 10.1093/jxb/erq468.

**Caleosin-like protein**

Yun Young Kim, Kwang Wook Jung, Kyoung Shin Yoo, Ji Ung Jeung, Jeong Sheop Shin (2011) A stress-responsive caleosin-like protein, atclo4, acts as a negative regulator of ABA responses in Arabidopsis. Plant Cell Physiol 52(5): 874–884. doi:10.1093/pcp/pcr039

**Callose synthase**

Estrella Luna, Victoria Pastor, Jérôme Robert, Victor Flors, Brigitte Mauch-Mani, Jurriaan Ton (2011) Callose deposition: a multifaceted plant defense response. Mol Plant-Microbe Interact 24(2): 183–193. doi:10.1094 / MPMI -07-10-0149

**CAP superfamily protein**

Pei-Shan Chien, Hong Gil Nam, Yet-Ran Chen (2015) A salt-regulated peptide derived from the CAP superfamily protein negatively regulates salt-stress tolerance in *Arabidopsis.* J Exp Bot 66(17): 5301–5313. doi:10.1093/jxb/erv263

**Carnitine operon (synthesis)**

Oney-Birol S (2019) Exogenous L-carnitine promotes plant growth and cell division by mitigating genotoxic damage of salt stress. Nature Sci Rep 9: 17229. https://doi.org/10.1038/s41598-019-53542-2

**CASP-like protein**

Jinghua Yang, Changqing Ding, Baochen Xu, Cuiting Chen, Reena Narsai, Jim Whelan, Zhongyuan Hu, Mingfang Zhang (2015) A Casparian strip domain-like gene, CASPL, negatively alters growth and cold tolerance. Sci Rep 5: 14299. DOI: 10.1038/srep14299

**CAX-interacting protein 4**

Bu Y, Fu W, Chen J, Takano T, Liu S (2021) Description of AtCAX4 in Response to Abiotic Stress in Arabidopsis. Int J Mol Sci 22: 856. https://doi.org/10.3390/ijms22020856

**Cell wall invertase**

MJ Nishanth, SA Sheshadri, Sudarshan Singh Rathore, S Srinidhi, Bindu Simon (2018) Expression analysis of Cell wall invertase under abiotic stress conditions influencing specialized metabolism in *Catharanthus roseus*. Sci Rep 8: 15059. DOI:10.1038/s41598-018-33415-w

**Chalcone synthase**

Dao TT, Linthorst HJ, Verpoorte R (2011) Chalcone synthase and its functions in plant resistance. Phytochem Rev 10(3): 397-412. doi: 10.1007/s11101-011-9211-7.

**Chloroplast heat shock protein 70-2**

Saeed ul Haq, Abid Khan, Muhammad Ali, Abdul Mateen Khattak, Wen-Xian Gai, Huai-Xia Zhang, Ai-Min Wei, Zhen-Hui Gong (2019). Heat shock proteins: dynamic biomolecules to counter plant biotic and abiotic stresses. Int J Mol Sci 20: 5321. doi:10.3390/ijms20215321

**Cold acclimation protein**

**Cold induced protein**

UniProt O65639 · CSP1_ARATH

Chinnusamy V, Zhu JK, Sunkar R (2010) Gene regulation during cold stress acclimation in plants. Methods Mol Biol 639: 39-55. doi: 10.1007/978-1-60761-702-0_3

**Cold-regulated 413-plasma membrane**

[Breton G](https://www.uniprot.org/uniprotkb?query=lit_author:%22Breton%20G.%22), [Danyluk J](https://www.uniprot.org/uniprotkb?query=lit_author:%22Danyluk%20J.%22), [Charron J-BF](https://www.uniprot.org/uniprotkb?query=lit_author:%22Charron%20J.-B.F.%22), [Sarhan F](https://www.uniprot.org/uniprotkb?query=lit_author:%22Sarhan%20F.%22) (2003) Expression profiling and bioinformatic analyses of a novel stress-regulated multispanning transmembrane protein family from cereals and Arabidopsis. Plant Physiol 132(1) 64–74. <https://doi.org/10.1104/pp.102.015255>

**Cold-regulated protein 27**

Peng Wang, Xuan Cui, Chunsheng Zhao, Liyan Shi, Guowei Zhang, Fenglong Sun, Xiaofeng Cao, Li Yuan, Qiguang Xie, Xiaodong Xu (2017) COR27 and COR28 encode nighttime repressors integrating Arabidopsis circadian clock and cold response. J Integr Plant Biol 59(2): 78–85. doi: 10.1111/jipb.12512

**Cold shock domain protein**

Kentaro Sasaki, Ryozo Imai (2012) Pleiotropic roles of cold shock domain proteins in plants. Front Plant Sci 2: 116. doi: 10.3389/fpls.2011.00116

**Copalyl diphosphate synthase**

Ai-Xia Cheng, Yong-Gen Lou, Ying-Bo Mao, Shan Lu, Ling-Jian Wang, Xiao-Ya Chen (2007) Plant terpenoids: biosynthesis and ecological functions. J Integr Plant Biol 49(2): 179−186.

**Copper ion binding protein**

**Copper transport protein**

**Copper-transporting ATPase**

Yruela I (2009) Copper in plants: acquisition, transport and interactions. Func Plant Biol 36: 409-430.

**Coronatine insensitive 1**

Xie DX, Feys BF, James S, Nieto-Rostro M, Turner JG (1998) COI1: an Arabidopsis gene required for jasmonate-regulated defense and fertility. Science. 280(5366): 1091-1094. doi: 10.1126/science.280.5366.1091.

**Cyanate hydratase**

KEGG EC 4.2.1.104. Cyanate hydratase or cyanase lyase catalyses the reaction of cyanate with bicarbonate to produce ammonia and carbon dioxide, allowing the host organisms to overcome the toxicity of environmental cyanate. The enzyme is also known as cyanate hydrolase.

**CYCLOP**S

Yano K, Yoshida S, Müller J, Singh S, Banba M, Vickers K, Markmann K, White C, Schuller B, Sato S, Asamizu E, Tabata S, Murooka Y, Perry J, Wang TL, Kawaguchi M, Imaizumi-Anraku H, Hayashi M, Parniske M (2008) CYCLOPS, a mediator of symbiotic intracellular accommodation. Proc Natl Acad Sci USA 105(51): 20540-20545. doi: 10.1073/pnas.0806858105

**Cystathionine gamma-synthase**

Hagai Cohen, Hadasa Israeli, Ifat Matityahu, Rachel Amir (2014) Seed-specific expression of a feedback-insensitive form of CYSTATHIONINE-g-SYNTHASE in Arabidopsis stimulates metabolic and transcriptomic responses associated with desiccation stress. Plant Physiol 166: 1575–1592. www.plantphysiol.org/cgi/doi/10.1104/pp.114.246058

**Cysteine and histidine-rich domain-containing protein RAR1**

Paul R Muskett, Katherine Kahn, Mark J Austin, Lisa J Moisan, Ari Sadanandom, Ken Shirasu, Jonathan DG Jones, Jane E Parker (2002) Arabidopsis *RAR1* exerts rate-limiting control of *R* gene–mediated defenses against multiple pathogens. Plant Cell 14: 979–992. www.plantcell.org/cgi/doi/10.1105/tpc.001040.

**Cysteine proteinase inhibitors**

Solomon M, Belenghi B, Delledonne M, Menachem E, Levine A (1999) The involvement of cysteine proteases and protease inhibitor genes in the regulation of programmed cell death in plants. Plant Cell 11(3): 431-444. doi: 10.1105/tpc.11.3.431

**Cysteine-rich venom protein**

**Snake venom serine proteinase**

Member of CAP protein superfamily

Takashi Tadokoro, Cassandra M Modahl, Katsumi Maenaka, Narumi Aoki-Shioi (2020) Cysteine-rich secretory proteins (CRISPs) from venomous snakes: an overview of the functional diversity in a large and underappreciated superfamily. Toxins 12: 175. doi:10.3390/toxins12030175

**Cytochrome P450**

Pandian BA, Sathishraj R, Djanaguiraman M, Prasad PVV, Jugulam M (2020) Role of cytochrome P450 enzymes in plant stress response. Antioxidants (Basel) 9(5): 454. doi: 10.3390/antiox9050454.

**Cytochrome P450 family cinnamate 4-hydroxylase**

Dolly A Bell-Lelong, Joanne C Cusumano, Knut Meyer, Clint Chapple (1997) Cinnamate-4-hydroxylase expression in Arabidopsis Regulation in response to development and the environment. Plant Physiol 113: 729-738.

**DAD1**

Yan Q, Si J, Cui X, Peng H, Jing M, Chen X, Xing H, Dou D (2019) GmDAD1, a conserved Defender Against Cell Death 1 (DAD1) from soybean, positively regulates plant resistance against *Phytophthora* pathogens. Front Plant Sci 10: 107. doi: 10.3389/fpls.2019.00107

**DCD proteins**

Raimund Tenhaken, Tobias Doerks, Peer Bork (2005) DCD – a novel plant specific domain in proteins involved in development and programmed cell death. [BMC Bioinform](https://link.springer.com/journal/12859) 6: 169. http://www.biomedcentral.com/1471-2105/6/169

**Defensin**

Sathoff AE, Samac DA (2019) Antibacterial activity of plant defensins. Mol Plant-microbe Interact 32(5): 507-514. doi: 10.1094/MPMI-08-18-0229-CR

**Dehydration-induced 19**

Milla MA, Townsend J, Chang IF, Cushman JC (2006) The Arabidopsis AtDi19 gene family encodes a novel type of Cys2/His2 zinc-finger protein implicated in ABA-independent dehydration, high-salinity stress and light signaling pathways. Plant Mol Biol 61(1-2): 13-30. doi: 10.1007/s11103-005-5798-7.

**Dehydration responsive element binding factor protein**

Pradeep K Agarwal, Kapil Gupta, Sergiy Lopato, Parinita Agarwal (2017) Dehydration responsive element binding transcription factors and their applications for the engineering of stress tolerance. J Exp Bot 68( 9): 2135–2148. doi:10.1093/jxb/erx118

**Dehydrin**

Sun Z, Li S, Chen W, Zhang J, Zhang L, Sun W, Wang Z (2021) Plant dehydrins: expression, regulatory networks, and protective roles in plants challenged by abiotic stress. Int J Mol Sci 22: 12619. <https://doi.org/10.3390/ijms> 222312619

**Dehydrogenase/reductase SDR family member**

Hyong Woo Choi, Byung Gil Lee, Nak Hyun Kim, Yong Park, Chae Woo Lim, Hyun Kyu Song, Byung Kook Hwang (2008) A role for a menthone reductase in resistance against microbial pathogens in plants. Plant Physiol 148: 383–340. [www.plantphysiol.org/cgi/doi/10.1104/pp.108.119461](http://www.plantphysiol.org/cgi/doi/10.1104/pp.108.119461)

San-Gwang Hwang, Nai-Chun Lin, Yi-Yun Hsiao, Ching-Hsuan Kuo, Pi-Fang Chang, Wen-Ling Deng, Ming-Hau Chiang, Hwei-Ling Shen, Chao-Ying Chen, Wan-Hsing Cheng (2011) The Arabidopsis short-chain dehydrogenase/reductase 3, an ABSCISIC ACID DEFICIENT 2 homolog, is involved in plant defense responses but not in ABA biosynthesis. Plant Physiol Biochem 51: 63-73. doi:10.1016/j.plaphy.2011.10.013

**Dehydroquinate dehydratase/shikimate dehydrogenase**

Lefevere H, Bauters L, Gheysen G (2020) Salicylic acid biosynthesis in plants. Front. Plant Sci. 11: 338. doi: 10.3389/fpls.2020.00338

**Detoxification protein**

Li L, He Z, Pandey GK, Tsuchiya T, Luan S (2002) Functional cloning and characterization of a plant efflux carrier for multidrug and heavy metal detoxification. J Biol Chem 277(7): 5360-5368. doi: 10.1074/jbc.M108777200

**Dirigent protein**

**Disease resistance-responsive (dirigent-like protein) family protein**

Candelas Paniagua, Anna Bilkova, Phil Jackson, Siarhei Dabravolski, Willi Riber, Vojtech Didi, Josef Houser, Nora Gigli-Bisceglia, Michaela Wimmerova, Eva Budínská, Thorsten Hamann, Jan Hejatko (2017) Dirigent proteins in plants: modulating cell wall metabolism during abiotic and biotic stress exposure. J Exp Bot 68(13): 3287–3301. doi:10.1093/jxb/erx141

**Divalent-cation tolerance protein CutA**

Khaled A Selim, Lorena Tremi, Clara Marco-Marin, Vikram Alva, Javier Espinosa, Asuncion Contreras, Marcus D Hartmann, Karl Forchhammer, Vicente Rubio (2021) Functional and structural characterization of PII-like protein CutA does not support involvement in heavy metal tolerance and hints at a small-molecule carrying/signaling role. FEBS J 288: 1142–1162. doi:10.1111/febs.15464

**DJ-1**

Xiang Ming Xu, Hong Lin, Jodi Maple, Benny Björkblom, Guido Alves, Jan Petter Larsen, Simon Geir Møller (2010) The Arabidopsis DJ-1a protein confers stress protection through cytosolic SOD activation. J Cell Sci 123: 1644-1651. doi:10.1242/jcs.063222

**DNA damage-inducible protein 1**

**DNA damage-binding protein**

Wang B, Wang G and Zhu S (2020) DNA damage inducible protein 1 is involved in cold adaption of harvested cucumber fruit. Front Plant Sci 10: 1723. doi: 10.3389/fpls.2019.01723

**DnaJ**

**DnaJ heat shock amino-terminal domain protein**

Rosita Grijalva-Mañaya, Carmen Dorca-Fornella, Wladimir Enríquez-Villacresesa, Gabriela Miño-Castroa, Ricardo Olivab, Valeria Ochoaa, Karina Proaño-Tumaa, Vinicio Armijos-Jaramillo (2019) DnaJ molecules as potential effectors in *Meloidogyne arenaria*. An unexplored group of proteins in plant parasitic nematodes. Comm Integr Biol 12 (1): 151–161. <https://doi.org/10.1080/19420889.2019.1676138>

**Early responsive dehydration stress ERD4**

Guofan Wu, Nongfu Tian, Fawen She, Aohua Cao, Wangze Wu, Sheng Zheng, Ning Yang (2022) Characteristics analysis of *Early Responsive to Dehydration* genes in *Arabidopsis thaliana* (*AtERD*). Plant Signal Behav 2: 2105021. doi: 10.1080/15592324.2022.2105021

**Early responsive to dehydration 15 (ERD15)**

Murilo S Alves, Elizabeth PB Fontes, Luciano G Fietto (2011) EARLY RESPONSIVE to DEHYDRATION 15, a new transcription factor that integrates stress signaling pathways. Plant Signal Behav 6(12): 1993-1996. DOI: 10.4161/psb.6.12.18268

**Elicitor-responsive protein**

Kim CY, Koo YD, Jin JB, Moon BC, Kang CH, Kim ST, Park BO, Lee SY, Kim ML, Hwang I, Kang KY, Bahk JD, Lee SY, Cho MJ (2003) Rice C2-domain proteins are induced and translocated to the plasma membrane in response to a fungal elicitor. Biochemistry 42(40): 11625-11633. doi: 10.1021/bi034576n

# Elongation factor 1-alpha (eEF1A1)

Oleksandra Novosylna, Ewelina Jurewicz, Nikolay Pydiura, Agnieszka Goral, Anna Filipek, Boris Negrutskii, Anna El'skaya (2015) Translation elongation factor eEF1A1 is a novel partner of a multifunctional protein Sgt1. Biochimie [119](https://www.sciencedirect.com/journal/biochimie/vol/119/suppl/C): 137-145. <https://doi.org/10.1016/j.biochi.2015.10.026>

**Enhanced disease resistance 2**

Sonja Vorwerk, Celine Schiff, Marjorie Santamaria, Serry Koh, Marc Nishimura, John Vogel, Chris Somerville, Shauna Somerville (2007) *EDR2* negatively regulates salicylic acid-based defenses and cell death during powdery mildew infections of *Arabidopsis thaliana.* BMC Plant Biol 7: 35. doi:10.1186/1471-2229-7-35.

**ent-Kaurene synthase**

Toyomasu T et al. (2016) Characterization and evolutionary analysis of *ent-*kaurene synthase like genes from the wild rice species *Oryza rufipogon.* Biochem Biophys Res Comm 480(3): 402-408.

**ENTH/ANTH/VHS superfamily protein**

Yihong Feng, Takuma Hiwatashi, Naoki Minamino, Kazuo Ebine, Takashi Ueda (2022) Membrane trafficking functions of the ANTH/ENTH/VHS domain-containing proteins in plants. FEBS Lett 596: 2256–2268. doi:10.1002/1873-3468.14368

**Ethylene insensitive 3**

Xiang He, Jishan Jiang, Changquan Wang, Katayoon Dehesh (2017) ORA59 and EIN3 interaction couples jasmonate-ethylene synergistic action to antagonistic salicylic acid regulation of PDF expression. J Integr Plant Biol 59(4): 275–287. doi:10.1111/jipb.12524

**Ethylene receptor**

# Kazan K (2015) Diverse roles of jasmonates and ethylene in abiotic stress tolerance. Trends Plant Sci [20 (4):](https://www.cell.com/trends/plant-science/issue?pii=S1360-1385(14)X0016-6) 219-229. DOI:<https://doi.org/10.1016/j.tplants.2015.02.001>

**Eukaryotic translation initiation factors**

**eIF4E**

Aiming Wang, Sowmya Krishnaswamy (2012) Eukaryotic translation initiation factor 4E-mediated recessive resistance to plant viruses and its utility in crop improvement. Mol Plant Pathol 13(7): 795–803. DOI: 10.1111/J.1364-3703.2012.00791.X

**eIF5A**

Liuqiang Wang, Chenxi Xu, Chao Wang, Yucheng Wang (2012) Characterization of a eukaryotic translation initiation factor 5A homolog from *Tamarix androssowii* involved in plant abiotic stress tolerance. BMC Plant Biol 12: 118. http://www.biomedcentral.com/1471-2229/12/118

**Exordium-like1**

Florian Schröder, Janina Lisso, Carsten Müssig, (2012) Expression pattern and putative function of EXL1 and homologous genes in Arabidopsis. Plant Signal Behav 7(1): 1–6. <https://doi.org/10.4161/psb.7.1.18369>

**Expansin**

Delia A. Narváez-Barragán, Omar E. Tovar-Herrera, Lorenzo Segovia, Mario Serrano, Claudia Martinez-Anaya (2020) Expansin-related proteins: biology, microbe–plant interactions and associated plant-defense responses. Microbiol 166: 1007–1018.

**FAD-binding berberine family protein**

Federica Locci, Manuel Benedetti, Daniela Pontiggia, Matteo Citterico, Claudio Caprari, Benedetta Mattei, Felice Cervone, Giulia De Lorenzo (2019) An Arabidopsis berberine bridge enzyme-like protein specifically oxidizes cellulose oligomers and plays a role in immunity. Plant J 98: 540–554. doi: 10.1111/tpj.14237

**Fer-like iron deficiency-induced transcription factor**

Bauer P, Ling HQ, Guerinot ML(2007) FIT, the FER-LIKE IRON DEFICIENCY INDUCED TRANSCRIPTION FACTOR in Arabidopsis. Plant Physiol Biochem 45(5): 260-261. doi: 10.1016/j.plaphy.2007.03.006

John H Herlihy, Terri A Long, John M McDowell (2020) Iron homeostasis and plant immune responses: Recent insights and translational implications. J Biol Chem 295(39): 13444–13457. DOI 10.1074/jbc.REV120.010856

**Ferritin**

John H Herlihy, Terri A Long, John M McDowell (2020) Iron homeostasis and plant immune responses: Recent insights and translational implications. J Biol Chem 295(39): 13444–13457. DOI 10.1074/jbc.REV120.010856

**Flavonoid 3'-hydroxylase**

**Flavonoid 3',5'-hydroxylase**

**Flavonoid 3'-monooxygenase**

Treutter D (2006) Significance of flavonoids in plant resistance: a review. Environ Chem Lett 4(4): 147–157. <https://doi.org/10.1007/s10311-006-0068-8>

Sabrina Chin, Carolyn A Behm, Ulrike Mathesius (2018) Functions of flavonoids in plant–nematode interactions. Plants 7: 85. doi:10.3390/plants7040085

**Freezing-induced 1**

Thomashow MF (1999) Plant cold acclimation: freezing tolerance genes and regulatory mechanisms. Annu Rev Plant Physiol Plant Mol Biol 50: 571–599.

**Gamma-aminobutyric acid receptor subunit gamma-2**

Guo Z, Gong J, Luo S, Zuo Y, Shen Y (2023) Role of gamma-aminobutyric acid in plant defense response. Metabolites 13: 741. <https://doi.org/10.3390/> metabo13060741

**Germacrene C/D synthase**

[Claus O Schmidt](https://onlinelibrary.wiley.com/authored-by/Schmidt/Claus+O.), [Harro J Bouwmeester](https://onlinelibrary.wiley.com/authored-by/Bouwmeester/Harro+J.), [Stephan Franke](https://onlinelibrary.wiley.com/authored-by/Franke/Stephan), [Wilfried A König](https://onlinelibrary.wiley.com/authored-by/K%C3%B6nig/Wilfried+A.) (1999) Mechanisms of the biosynthesis of sesquiterpene enantiomers (+)- and (−)-germacrene D in *Solidago canadensis.* Chirality 11(5-6): 353-362. [https://doi.org/10.1002/(SICI)1520-636X(1999)11:5/6<353::AID-CHIR2>3.0.CO;2-L](https://doi.org/10.1002/(SICI)1520-636X(1999)11:5/6%3c353::AID-CHIR2%3e3.0.CO;2-L)

**Germin-like protein**

Dunwell, J, Gibbings JG, Mahmood T, Saqlan Naqvi SM (2008) Germin and germin-like proteins: evolution, structure, and function. Crit Rev Plant Sci 27(5): 342-375. doi: <https://doi.org/10.1080/07352680802333938>.

**Globulin seed storage proteins**

Kesari P, Neetu, Sharma A, Katiki M, Kumar P, Gurjar BR, Tomar S, Sharma AK, Kumar P (2017) Structural, functional and evolutionary aspects of seed globulins. Protein Pept Lett 24(3): 267-277. doi: 10.2174/0929866523666161220112641

**Haloacid dehalogenase-like hydrolase**

Du Z, Deng S, Wu Z, Wang C (2021) Genome-wide analysis of haloacid dehalogenase genes reveals their function in phosphate starvation responses in rice. PLoS ONE 16(1): e0245600. <https://doi.org/10.1371/journal.pone.0245600>

**Harpin**

Krause M, Durner J (2004) Harpin inactivates mitochondria in *Arabidopsis* suspension cells. Mol Plant-Microbe Interact 17(2): 131–139. *Part of Mt oxidative burst.*

**Heat shock 70 kDa protein**

**70kDa heat shock protein**

**HSP70**

**Heat shock factor-binding protein 1**

**Heat shock transcription protein**

Miroslav Berka, Romana Kopecká, Veronika Berková, Břetislav Brzobohatý, Martin Černý (2022) Regulation of heat shock proteins 70 and their role in plant immunity. J Exp Bot 73(7): 1894–1909. <https://doi.org/10.1093/jxb/erab549>

Saeed ul Haq, Abid Khan, Muhammad Ali, Abdul Mateen Khattak, Wen-Xian Gai, Huai-Xia Zhang, Ai-Min Wei, Zhen-Hui Gong (2019). Heat shock proteins: dynamic biomolecules to counter plant biotic and abiotic stresses. Int J Mol Sci 20: 5321. doi:10.3390/ijms20215321

**Heat shock protein 90**

Yasuhiro Kadota, Ken Shirasu (2012) The HSP90 complex of plants. Biochim Biophys Acta 1823: 689-697. doi:10.1016/j.bbamcr.2011.09.016

**Heat stress transcription factor A**

Samtani H, Sharma A, Khurana P (2022) Wheat ocs-element binding factor 1 enhances thermotolerance by modulating the heat stress response pathway. Front Plant Sci 13: 914363.

doi: 10.3389/fpls.2022.914363

**Heat stress transcription factor B**

Guo M, Liu J-H, Ma X, Luo D-X, Gong Z-H, Lu M-H (2016) The plant heat stress transcription factors (HSFs): structure, regulation, and function in response to abiotic stresses. Front Plant Sci 7: 114. doi: 10.3389/fpls.2016.00114

**Heavy metal transport/detoxification superfamily protein**

**Heavy metal-associated protein**

Zhao F-J, Tang Z, Song J-J, Huang X-Y, Wang P (2022) Toxic metals and metalloids: uptake, transport, detoxification, phytoremediation, and crop improvement for safer food. Mol Plant 15: 27–44. https://doi.org/10.1016/j.molp.2021.09.016

**Histone H1**

Arsheed H Sheikh, Kashif Nawaz, Naheed Tabassum, Marilia Almeida-Trapp, Kiruthiga G Mariappan, Hanna Alhoraibi, Naganand Rayapuram, Manuel Aranda, Martin Groth, Heribert Hirt (2023) Linker histone H1 modulates defense priming and immunity in plants. Nucl Acids Res 51(9): 4252–4265. https://doi.org/10.1093/nar/gkad106

**Homoserine/homoserine lactone efflux protein**

Sebastian T Schenk, Casandra Hernández-Reyes, Birgit Samans, Elke Stein, Christina Neumann, Marek Schikora, Michael Reichelt, Axel Mithöfer, Annette Becker, Karl-Heinz Kogel, Adam Schikora (2014) N-acyl-homoserine lactone primes plants for cell wall reinforcement and induces resistance to bacterial pathogens via the salicylic acid/oxylipin pathway. Plant Cell 26: 2708–2723. [www.plantcell.org/cgi/doi/10.1105/tpc.114.126763](http://www.plantcell.org/cgi/doi/10.1105/tpc.114.126763)

**Hopanoid-associated sugar epimerase**

Belin BJ, Busset N, Giraud E, Molinaro A, Silipo A, Newman DK (2018) Hopanoid lipids: from membranes to plant-bacteria interactions. Nat Rev Microbiol 16(5): 304-315. doi: 10.1038/nrmicro.2017.173.

**HR-like lesion-inducing protein**

Liang Zhou, Ming-Yan Cheung, Man-Wah Li, Yaping Fu, Zongxiu Sun, Sai-Ming Sun, Hon-Ming Lam (2010) Rice Hypersensitive Induced Reaction Protein 1 (OsHIR1) associates with plasma membrane and triggers hypersensitive cell death. BMC Plant Biol 10: 290. http://www.biomedcentral.com/1471-2229/10/290

**HSP20-like**

**HSP20-like chaperones superfamily protein**

Ji XR, Yu YH, Ni PY, Zhang GH, Guo DL (2019) Genome-wide identification of small heat-shock protein (HSP20) gene family in grape and expression profile during berry development. BMC Plant Biol 19(1): 433. doi: 10.1186/s12870-019-2031-4

**htpG**

Song H, Zhao R, Fan P, Wang X, Chen X, Li Y (2009) Overexpression of AtHsp90.2, AtHsp90.5 and AtHsp90.7 in *Arabidopsis thaliana* enhances plant sensitivity to salt and drought stresses. Planta 229(4): 955-964. doi: 10.1007/s00425-008-0886-y

**Huntington interacting protein K**

Das S, Bhattacharyya NP (2016) Huntingtin interacting protein HYPK is a negative regulator of heat shock response and is downregulated in models of Huntington's Disease. Exp Cell Res 343(2): 107-117. doi: 10.1016/j.yexcr.2016.03.021

**HVA22**

Alex Brands, Tuan-hua David Ho (2022) Function of a plant stress-induced gene, *HVA22*. Synthetic enhancement screen with its yeast homolog reveals its role in vesicular traffic. Plant Physiol 130: 1121–1131.

**Hydrogen peroxide-induced 1**

Steven Vandenabeele, Katrien Van Der Kelen, James Dat, Ilya Gadjev, Tom Boonefaes, Stijn Morsa, Pieter Rottiers, Luit Slooten, Marc Van Montagu, Marc Zabeau, Dirk Inze, Frank Van Breusegem (2003) A comprehensive analysis of hydrogen peroxide-induced gene expression in tobacco. Proc Natl Acad Sci USA 100(26): 16113–16118. [www.pnas.org_cgi_doi_10.1073_pnas.2136610100](http://www.pnas.org_cgi_doi_10.1073_pnas.2136610100)

**Hydroxyacylglutathione hydrolase**

Mirza Hasanuzzaman, Kamrun Nahar, Taufika Islam Anee, Masayuki Fujita (2017) Glutathione in plants: biosynthesis and physiological role in environmental stress tolerance. Physiol Mol Biol Plants 23(2): 249–268. DOI 10.1007/s12298-017-0422-2

**Hydroxycinnamoyl-CoA shikimate/quinate hydroxycinnamoyl transferase**

Guan-Feng Wang, Yijian He, Renee Strauch, Bode A Olukolu, Dahlia Nielsen, Xu Li, Peter J Balint-Kurti (2010) Maize homologs of Hydroxycinnamoyltransferase, a key enzyme in lignin biosynthesis, bind the nucleotide binding leucine-rich repeat Rp1 proteins to modulate the defense response. Plant Physiol 169: 2230–2243. www.plantphysiol.org/cgi/doi/10.1104/pp.15.00703

**Hypersensitive-induced response protein**

Liang Zhou, Ming-Yan Cheung, Man-Wah Li, Yaping Fu, Zongxiu Sun, Sai-Ming Sun, Hon-Ming Lam (2010) Rice Hypersensitive Induced Reaction Protein 1 (OsHIR1) associates with plasma membrane and triggers hypersensitive cell death. BMC Plant Biol 10: 290. http://www.biomedcentral.com/1471-2229/10/290

**Hypoxia-induced or -responsive-protein**

Huh SU (2021) New function of Hypoxia-responsive unknown protein in enhanced resistance to biotic stress. Plant Signal Behav 16(3): 1868131. DOI:10.1080/15592324.2020.1868131

**Invertase/pectin methylesterase inhibitor superfamily protein**

Coculo D, Lionetti V (2022) The plant invertase/pectin methylesterase inhibitor superfamily. Front Plant Sci 13: 863892. doi: 10.3389/fpls.2022.863892

**Isoflavone 4'-O-methyltransferase**

**Isoflavone-7-O-methyltransferase**

**Isoflavone reductase**

Chang-Jun Liu, Bettina E Deavours, Stephane B Richard, Jean-Luc Ferrer, Jack W. Blount, David Huhman, Richard A Dixon, Joseph P Noela, (2006) structural basis for dual functionality of isoflavonoid o-methyltransferases in the evolution of plant defense responses. Plant Cell 18: 3656–3669. www.plantcell.org/cgi/doi/10.1105/tpc.106.041376

**Isopentenyl-diphosphate delta-isomerase**

González-Cabanelas D, Wright LP, Paetz C, Onkokesung N, Gershenzon J, Rodríguez-Concepción M, Phillips MA (2015) The diversion of 2-C-methyl-D-erythritol-2,4-cyclodiphosphate from the 2-C-methyl-D-erythritol 4-phosphate pathway to hemiterpene glycosides mediates stress responses in *Arabidopsis thaliana*. Plant J 82(1): 122-137. doi: 10.1111/tpj.12798

Nawaporn Onkokesung, Michael Reichelt, Louwrance P Wright, Michael A Phillips, Jonathan Gershenzon, Marcel Dicke (2019) The plastidial metabolite 2‐C‐methyl‐D‐erythritol‐2,4‐cyclodiphosphate modulates defence responses against aphids. Plant Cell Environ. 42: 2309–2323. DOI: 10.1111/pce.13538

**Farnesyl pyrophosphate synthase**

**Geranylgeranyl pyrophosphate synthase**

**Geranylgeranyl diphosphate reductase, chloroplastic**

Kai Ament, Chris C Van Schie, Harro J Bouwmeester, Michel A Haring, Robert C Schuurink (2006) Induction of a leaf specific geranylgeranyl pyrophosphate synthase and emission of (*E,E*)-4,8,12-trimethyltrideca-1,3,7,11-tetraene in tomato are dependent on both jasmonic acid and salicylic acid signaling pathways. Planta 224: 1197–1208. DOI 10.1007/s00425-006-0301-5

**Jasmonate O-methyltransferase**

Hak Soo Seo, Jong Tae Song, Jong-Joo Cheong, Yong-Hwan Lee, Yin-Won Lee, Ingyu Hwang, Jong Seob Lee, Yang Do Choi (2001) Jasmonic acid carboxyl methyltransferase: A key enzyme for jasmonate-regulated plant responses. Proc Natl Acad Sci USA 98(8): 4788–4793. [www.pnas.orgycgiydoiy10.1073ypnas.081557298](http://www.pnas.orgycgiydoiy10.1073ypnas.081557298)

**Jasmonate-ZIM-domain protein (JAZ)**

Oblessuc PR, Obulareddy N, DeMott L, Matiolli CC, Thompson BK, Melotto M (2020) JAZ4 is involved in plant defense, growth, and development in Arabidopsis. Plant J 101(2): 371-383. doi: 10.1111/tpj.14548.

Pauwels L, Goosshens A (2011) The JAZ proteins: a crucial interface in the jasmonate signaling cascade. Plant Cell 23: 3089–3100. www.plantcell.org/cgi/doi/10.1105/tpc.111.089300

**Laccase**

Wang J, Feng J, Jia W, Chang S, Li S, Li Y (2015) Lignin engineering through laccase modification: a promising field for energy plant improvement. Biotechnol Biofuels 8: 145. doi: 10.1186/s13068-015-0331-y

**Late embryogenesis-abundant proteins**

**LEA hydroxyproline-rich glycoprotein family**

Bao Y, Song W-M, Pan J, Jiang C-M, Srivastava R, Li B, et al. (2016) Overexpression of the NDR1/HIN1-like gene NHL6 modifies seed germination in response to abscisic acid and abiotic stresses in Arabidopsis. PLoS ONE 11(2): e0148572. doi:10.1371/journal.pone.0148572

**LEA5**

Karpinska B, Razak N, Shaw DS, Plumb W, Van De Slijke E, Stephens J, De Jaeger G, Murcha MW, Foyer CH (2022) Late Embryogenesis Abundant (LEA)5 regulates translation in mitochondria and chloroplasts to enhance growth and stress tolerance. Front Plant Sci 13: 875799. doi: 10.3389/fpls.2022.87579

**LEA Grp 3**

Boswell LC, Menze MA, Hand SC (2014) Group 3 late embryogenesis abundant proteins from embryos of *Artemia franciscana*: structural properties and protective abilities during desiccation. Physiol Biochem Zool 2014 Sep-Oct;87(5):640-651. doi: 10.1086/676936

**LEA14**

[Meizhen Wang](https://pubmed.ncbi.nlm.nih.gov/?term=Wang%20M%5BAuthor%5D), [Ping Li](https://pubmed.ncbi.nlm.nih.gov/?term=Li%20P%5BAuthor%5D), [Cong Li](https://pubmed.ncbi.nlm.nih.gov/?term=Li%20C%5BAuthor%5D), [Yanlin Pan](https://pubmed.ncbi.nlm.nih.gov/?term=Pan%20Y%5BAuthor%5D), [Xiyuan Jiang](https://pubmed.ncbi.nlm.nih.gov/?term=Jiang%20X%5BAuthor%5D), [Dengyun Zhu](https://pubmed.ncbi.nlm.nih.gov/?term=Zhu%20D%5BAuthor%5D), [Qian Zhao](https://pubmed.ncbi.nlm.nih.gov/?term=Zhao%20Q%5BAuthor%5D), [Jingjuan Yu](https://pubmed.ncbi.nlm.nih.gov/?term=Yu%20J%5BAuthor%5D) (2014) SiLEA14, a novel atypical LEA protein, confers abiotic stress resistance in foxtail millet. BMC Plant Biol 14: 290. doi: 10.1186/s12870-014-0290-7

**Lipid transfer protein**

Hang Gao, Kang Ma, Guojie Ji, Liying Pan, Qingfeng Zhou (2022) Lipid transfer proteins involved in plant–pathogen interactions and their molecular mechanisms. Mol Plant Pathol 23: 1815–1829. DOI: 10.1111/mpp.13264

**Loricrin**

Guo T, Wang X-W, Shan K, Sun W, Guo L-Y (2017) The Loricrin-like protein (LLP) of *Phytophthora infestans* is required for oospore formation and plant infection. Front. Plant Sci 8:142. doi: 10.3389/fpls.2017.00142

**Low temperature and salt responsive protein (RCI2A)**

Sivankalyani V, Geetha M, Subramanyam K, Girija S (2015) [Ectopic expression of Arabidopsis RCI2A gene contributes to cold tolerance in tomato.](https://pubmed.ncbi.nlm.nih.gov/25260337/) Transgenic Res 24(2): 237-251. doi: 10.1007/s11248-014-9840-x

Mitsuya S, Taniguchi M, Miyake H, Takabe T (2005) [Disruption of RCI2A leads to over-accumulation of Na+ and increased salt sensitivity in *Arabidopsis thaliana* plants.](https://pubmed.ncbi.nlm.nih.gov/16034593/) Planta 222(6): 1001-1009. doi: 10.1007/s00425-005-0043-9

**LSD1**

Dietrich RA, Richberg MH, Schmidt R, Dean C, Dangl JL (1997) A novel zinc finger protein is encoded by the Arabidopsis LSD1 gene and functions as a negative regulator of plant cell death. Cell 88(5): 685-694. doi: 10.1016/s0092-8674(00)81911-x

**LURP1-related**

Knoth C, Eulgem T (2008) The oomycete response gene LURP1 is required for defense against *Hyaloperonospora parasitica* in *Arabidopsis thaliana*. Plant J 55(1): 53-64. doi: 10.1111/j.1365-313X.2008.03486.x

**Major facilitator superfamily**

Carl R Simmons, Marcelo Fridlender, Pedro A Navarro, Nasser Yalpani (2003) A maize defense-inducible gene is a major facilitator superfamily member related to bacterial multidrug resistance efflux antiporters. Plant Mol Biol 52: 433–446.

**Maspardin**

Wang Fei-Bing, Wan Chen-Zhong, Niu Hao-Fei, Qi Ming-Yang, Li Gang, Zhang Fan, Hu Lai-Bao, Ye Yu-Xiu, Wang Zun-Xin, Pei Bao-Lei, Chen Xin-Hong, Yuan Cai-yon (2023) *OsMas1*, a novel maspardin protein gene, confers tolerance to salt and drought stresses by regulating ABA signaling in rice. J Integr Agric 22(2): 341–359. doi: 10.1016/j.jia.2022.08.077

**MATE efflux transporter**

Neha Upadhyay, Debojyoti Kar, Bhagyashri Deepak Mahajan, Sanchali Nanda, Rini Rahiman, Nimisha Panchakshari, Lavanya Bhagavatula, Sourav Datta (2019) The multitasking abilities of MATE transporters in plants. J Exp Bot 70(18). 4643–4656. doi:10.1093/jxb/erz246

**Membrane-associated salt-inducible protein**

(UniProt Q8W356_ORYSJ)

**Metacaspase**

Huh Su (2022) Evolutionary diversity and function of metacaspases in plants: similar to but not caspases. Int J Mol Sci 23: 4588. <https://doi.org/10.3390/ijms23094588>

**Metal tolerance protein**

Felipe K Ricachenevsky, Paloma K Menguer, Raul A Sperotto, Lorraine E Williams, Janette P Fett (2013) Roles of plant metal tolerance proteins (MTP) in metal storage and potential use in biofortification strategies Front Plant Sci 4: 144. doi: 10.3389/fpls.2013.00144

**Metallo-**β**-lactamase**

Guillermo Bahr, Lisandro J. González, Alejandro J. Vila (2021) Metallo-β-lactamases in the age of multidrug resistance: from structure and mechanism to evolution, dissemination and inhibitor design. Chem Rev 121(13): 7957–8094. doi:10.1021/acs.chemrev.1c00138

**Metallothionin**

Himanshu V Patankar, Ibtisam Al-Harrasi, Latifa Al Kharusi, Gerry Aplang Jana, Rashid Al-Yahyai, Ramanjulu Sunkar, Mahmoud W Yaish (2019) Overexpression of a *Metallothionein* 2A gene from date palm confers abiotic stress tolerance to yeast and *Arabidopsis thaliana*. Int J Mol Sci 20: 2871. doi:10.3390/ijms20122871

Joohyun Lee, Donghwan Shim, Won-Yong Song, Inhwan Hwang, Youngsook Lee (2004) Arabidopsis metallothioneins 2a and 3 enhance resistance to cadmium when expressed in *Vicia faba* guard cells. Plant Mol Biol 54: 805–815.

**Mevalonate kinase**

Cho SH, Tóth K, Kim D, Vo PH, Lin CH, Handakumbura PP, Ubach AR, Evans S, Paša-Tolić L, Stacey G (2022) Activation of the plant mevalonate pathway by extracellular ATP. Nat Commun 13(1): 450. doi: 10.1038/s41467-022-28150-w.

**MLO protein**

Piffanelli P, Zhou F, Casais C, Orme J, Jarosch B, Schaffrath U, Collins NC, Panstruga R, Schulze-Lefert P (2002) The barley MLO modulator of defense and cell death is responsive to biotic and abiotic stress stimuli. Plant Physiol 129: 1076-1085. DOI: [10.1104/pp.010954](https://doi.org/10.1104/pp.010954)

**Multidrug resistance protein Mdt**

**Multidrug resistance protein ABC transporter**

**Multidrug resistance-associated protein 2**

# [Markus Klein](https://pubmed.ncbi.nlm.nih.gov/?term=Klein+M&cauthor_id=16375897), [Bo Burla](https://pubmed.ncbi.nlm.nih.gov/?term=Burla+B&cauthor_id=16375897), [Enrico Martinoia](https://pubmed.ncbi.nlm.nih.gov/?term=Martinoia+E&cauthor_id=16375897) (2006) The multidrug resistance-associated protein (MRP/ABCC) subfamily of ATP-binding cassette transporters in plants. FEBS Lett 580(4): 1112-1122. doi: 10.1016/j.febslet.2005.11.056

**Mvp17**

Jiwoong Wi, Yeonju Na, Eunju Yang, Jung-Hyun Lee, Won-Joong Jeong, Dong-Woog Choi (2020) Arabidopsis AtMPV17, a homolog of mice MPV17, enhances osmotic stress tolerance. Physiol Mol Biol Plants 26(7): 1341–1348. <https://doi.org/10.1007/s12298-020-00834-x>

**N-Carbamoylputrescine amidase**

Piotrowski M, Janowitz T, Kneifel H (2003) Plant C-N hydrolases and the identification of a plant N-carbamoylputrescine amidohydrolase involved in polyamine biosynthesis. J Biol Chem 278(3): 1708-1712. doi: 10.1074/jbc.M205699200

Liu C, Atanasov KE, Arafaty N, Murillo E, Tiburcio AF, Zeier J, Alcázar R (2020) Putrescine elicits ROS-dependent activation of the salicylic acid pathway in Arabidopsis thaliana. Plant Cell Environ 43(11): 2755-2768. doi: 10.1111/pce.1387

**NADPH--cytochrome P450 reductase**

Yoshimi Yamamura, Ayaka Mabuchi (2020) Functional characterization of NADPH-cytochrome P450 reductase and cinnamic acid 4-hydroxylase encoding genes from *Scoparia dulcis* L. Bot Stud 61: 6. https://doi.org/10.1186/s40529-020-00284-4

**Naringenin,2-oxoglutarate 3-dioxygenase**

Wang Y, Shi Y, Li K, Yang D, Liu N, Zhang L, Zhao L, Zhang X, Liu Y, Gao L.; et al (2021) Roles of the 2-Oxoglutarate-Dependent Dioxygenase superfamily in the flavonoid pathway: a review of the functional diversity of F3H, FNS I, FLS, and LDOX/ANS. Molecules 26: 6745. <https://doi.org/10.3390/> molecules26216745

**NB-ARC domain disease resistance protein**

van Ooijen G, Mayr G, Kasiem MM, Albrecht M, Cornelissen BJ, Takken FL (2008) Structure-function analysis of the NB-ARC domain of plant disease resistance proteins. J Exp Bot 59(6): 1383-1397. doi: 10.1093/jxb/ern045.

**Negative regulator of resistance**

Chern M, Canlas PE, Fitzgerald HA, Ronald PC (2005) Rice NRR, a negative regulator of disease resistance, interacts with Arabidopsis NPR1 and rice NH1. Plant J 43(5): 623-635. doi: 10.1111/j.1365-313X.2005.02485.x

**Nematode resistance protein-like HSPRO1**

Murray SL, Ingle RA, Petersen LN, Denby KJ (2007) Basal resistance against *Pseudomonas syringae* in Arabidopsis involves WRKY53 and a protein with homology to a nematode resistance protein. Mol Plant-Microbe Interact 20: 1431-1438.

**(+)-Neomenthol dehydrogenase**

Choi HW, Lee BG, Kim NH, Park Y, Lim CW, Song HK, Hwang BK (2008) A role for a menthone reductase in resistance against microbial pathogens in plants. Plant Physiol 148(1): 383-401. doi: 10.1104/pp.108.119461

**Neutral invertase**

**Neutral/alkaline invertase**

Xiang L, Le Roy K, Bolouri-Moghaddam MR, Vanhaecke M, Lammens W, Rolland F, Van den Ende W (2011a) Exploring the neutral invertase-oxidative stress defense connection in *Arabidopsis thaliana.* J Exp Bot 62: 3849–3862. DOI:10.1093/jxb/err069

Addendum: Li Xiang, Yi Li, Filip Rolland, Wim Van den Ende (2011b) Neutral invertase, hexokinase and mitochondrial ROS homeostasis Emerging links between sugar metabolism, sugar signaling and ascorbate synthesis. Plant Signal Behav 6(10): 1567-1573. DOI: 10.4161/psb.6.10.17036

**Ninja**

Garcia ME, Lynch T, Peeters J, Snowden C, Finkelstein R (2008) Role of NINJA in root jasmonate signaling. Proc Natl Acad Sci USA 110(38): 15473–15478. [https://doi.org/10.1073/pnas.130791011](https://doi.org/10.1073/pnas.1307910110)

**Oberon**

Dasgupta U, Mishra GP, Dikshit HK, Mishra DC, Bosamia T, Roy A, Bhati J, Priti, Aski M, Kumar RR, Singh AK, Kumar A, Sinha SK, Chaurasia S, Praveen S, Nair RM (2021) Comparative RNA-Seq analysis unfolds a complex regulatory network imparting yellow mosaic disease resistance in mungbean [ (L.) R. Wilczek]. PLoS One 16(1): e0244593. doi: 10.1371/journal.pone.0244593

**OCS binding factor1**

Samtani H, Sharma A and Khurana P (2022) Wheat ocs-element binding factor 1 enhances thermotolerance by modulating the heat stress response pathway. Front Plant Sci 13: 914363.

doi: 10.3389/fpls.2022.914363

**Oxidation resistance protein**

Colombatti F, Mencia R, Garcia L, Mansilla N, Alemano S, Andrade AM, Gonzalez DH, Welchen E (2019) The mitochondrial oxidation resistance protein AtOXR2 increases plant biomass and tolerance to oxidative stress. J Exp Bot 70(12): 3177-3195. doi: 10.1093/jxb/erz147

**Oxidative stress 3**

Blanvillain R, Kim JH, Wu S, Lima A, Ow DW (2009). OXIDATIVE STRESS 3 is a chromatin-associated factor involved in tolerance to heavy metals and oxidative stress. Plant J 57(4): 654-665. doi: 10.1111/j.1365-313X.2008.03717.x

**Oxophytodienoate-reductase 3**

Wei Dong, Mengcheng Wang, Fei Xu, Taiyong Quan, Keqin Peng, Langtao Xiao, Guangmin Xia (2013) Wheat Oxophytodienoate Reductase gene TaOPR1 confers salinity tolerance via enhancement of abscisic acid signaling and reactive oxygen species scavenging. Plant Physiol 161: 1217–1228. [www.plantphysiol.org/cgi/doi/10.1104/pp.112.211854](http://www.plantphysiol.org/cgi/doi/10.1104/pp.112.211854)

**Oxysterol binding protein***

Anna O Avrova, Nawsheen Taleb, Veli-Matti Rokka, Jacqueline Heilbronn, Edward Campbell, Ingo Hein, Eleanor M Gilroy, Linda Cardle, John E Bradshaw, Helen E Stewart, Yasmina Jaufeerally Fakim, Gary Loake, Paul RJ Birch (2004) Potato oxysterol binding protein and cathepsin B are rapidly up-regulated in independent defence pathways that distinguish R gene-mediated and field resistances to *Phytophthora infestans.* Mol Plant Pathol 5(1): 45-56. DOI: 10.1046/J.1364-3703.2004.00205.X

**Ozone-responsive stress-related protein**

Nisar Ahmad Khan, Setsuko Komatsu, Hiroko Sawada, Mohammad-Zaman Nouri, Yoshihisa Kohno (2013) Analysis of proteins associated with ozone stress response in soybean

cultivars. Protein Pept Lett 20(10): 1144-1152. doi: 10.2174/09298665113209990003

**Patatin-like protein 4 & 5**

**Patatin**

Sylvain La Camera, Claudine Balagué, Cornelia Göbel, Pierrette Geoffroy, Michel Legrand, Ivo Feussner, Dominique Roby, Thierry Heitz (2009) The Arabidopsis Patatin-Like Protein 2 (PLP2) plays an essential role in cell death execution and differentially affects biosynthesis of oxylipins and resistance to pathogens. Mol Plant-Microbe Interac 22(4): 469–481. doi:10.1094 / MPMI -22-4-0469

**Pathogenesis-related transcriptional activator PTI6**

[Yong-Qiang Gu](https://pubmed.ncbi.nlm.nih.gov/?term=Gu%20YQ%5BAuthor%5D), [Mary C Wildermuth](https://pubmed.ncbi.nlm.nih.gov/?term=Wildermuth%20MC%5BAuthor%5D), [Suma Chakravarthy](https://pubmed.ncbi.nlm.nih.gov/?term=Chakravarthy%20S%5BAuthor%5D), [Ying-Tsu Loh](https://pubmed.ncbi.nlm.nih.gov/?term=Loh%20YT%5BAuthor%5D), [Caimei Yang](https://pubmed.ncbi.nlm.nih.gov/?term=Yang%20C%5BAuthor%5D), [Xiaohua He](https://pubmed.ncbi.nlm.nih.gov/?term=He%20X%5BAuthor%5D), Yu Han, [Gregory B Martin](https://pubmed.ncbi.nlm.nih.gov/?term=Martin%20GB%5BAuthor%5D) (2002) Tomato transcription factors PTI4, PTI5, and PTI6 activate defense responses when expressed in Arabidopsis. [Plant Cell](https://www.ncbi.nlm.nih.gov/pmc/articles/PMC150684/) 14(4): 817–831. doi:[10.1105/tpc.000794](https://doi.org/10.1105%2Ftpc.000794)

**Pathogen-related protein 1**

Sun Tae Kim, Seok Yu, Young Hyun Kang, Sang Gon Kim, Jae-Yean Kim, Sun-Hyung Kim,Kyu Young Kang (2008) The rice pathogen-related protein 10 (JIOsPR10) is induced by abiotic and biotic stresses and exhibits ribonuclease activity. Plant Cell Rep 27: 593–603.

DOI 10.1007/s00299-007-0485-6

**Peptide methionine sulfoxide reductase MsrA**

Delaye L, Becerra A, Orgel L, Lazcano A (2007) Molecular evolution of peptide methionine sulfoxide reductases (MsrA and MsrB): on the early development of a mechanism that protects against oxidative damage. J Mol Evol 64(1): 15-32. doi: 10.1007/s00239-005-0281-2

**Peptidyl-prolyl cis-trans isomerase**

Mokryakova MV, Pogorelko GV, Bruskin SA, Piruzian ES, Abdeeva IA (2014) The role of peptidyl-prolyl *cis*/*trans* isomerase genes of *Arabidopsis thaliana* in plant defense during the course of *Xanthomonas campestris* infection. Russ J Genet 50: 140–148. https://doi.org/10.1134/S1022795414020100

**Peroxidase**

Kaushik Das, Aryadeep Roychoudhury (2014) Reactive oxygen species (ROS) and response of antioxidants as ROS-scavengers during environmental stress in plants. Front Environ Sci 2: 53. doi: 10.3389/fenvs.2014.00053

Quiroga M, Guerrero C, Botella MA, Barceló A, Amaya I, Medina MI, Alonso FJ, de Forchetti SM, Tigier H, Valpuesta V (2000) A tomato peroxidase involved in the synthesis of lignin and suberin. Plant Physiol 122(4): 1119-1127. doi: 10.1104/pp.122.4.1119

**Peroxiredoxin**

König J, Baier M, Horling F, Kahmann U, Harris G, Schürmann P, Dietz KJ (2002) The plant-specific function of 2-Cys peroxiredoxin-mediated detoxification of peroxides in the redox-hierarchy of photosynthetic electron flux. Proc Natl Acad Sci USA 99(8): 5738-5743. Doi: 10.1073/pnas.072644999

p**fkB-like carbohydrate kinase family protein**

Jonathan Gilkerson, Juan Manuel Perez-Ruiz, Joanne Chory, Judy Callis (2012) The plastid-localized pfkB-type carbohydrate kinases FRUCTOKINASE-LIKE 1 and 2 are essential for growth and development of *Arabidopsis thaliana*. BMC Plant Biol 12: 102. http://www.biomedcentral.com/1471-2229/12/102

**Pirin**

Brunetti SC, Arseneault MKM, Gulick PJ. Characterization and expression of the *Pirin* gene family in *Triticum aestivum*. Genome 65(6): 349-362. doi: 10.1139/gen-2021-0094

**Pleiotropic drug resistance transporter**

**Pleiotropic drug resistance ABC transporter**

# [Anil Dahuja](https://onlinelibrary.wiley.com/authored-by/Dahuja/Anil), [Ranjeet R Kumar](https://onlinelibrary.wiley.com/authored-by/Kumar/Ranjeet+R.), [Akshay Sakhare](https://onlinelibrary.wiley.com/authored-by/Sakhare/Akshay), [Archana Watts](https://onlinelibrary.wiley.com/authored-by/Watts/Archana), [Bhupinder Singh](https://onlinelibrary.wiley.com/authored-by/Singh/Bhupinder), [Suneha Goswami](https://onlinelibrary.wiley.com/authored-by/Goswami/Suneha), [Archana Sachdev](https://onlinelibrary.wiley.com/authored-by/Sachdev/Archana), [Shelly Praveen](https://onlinelibrary.wiley.com/authored-by/Praveen/Shelly) (2021) Role of ATP-binding cassette transporters in maintaining plant homeostasis under abiotic and biotic stresses. Physiol Plantar 171(4): 785-801. <https://doi.org/10.1111/ppl.13302>

Nuruzzaman M, Zhang R, Cao HZ, Luo ZY (2014) Plant pleiotropic drug resistance transporters: transport mechanism, gene expression, and function. J Integr Plant Biol 56(8): 729-740. doi: 10.1111/jipb.12196

**Polyamine oxidase**

Wimalasekera R, Tebartz F, Scherer GFE (2011) Polyamines, polyamine oxidases and nitric oxide in development, abiotic and biotic stresses. [Plant Sci](https://www.sciencedirect.com/journal/plant-science) 181(5): 593-603. <https://doi.org/10.1016/j.plantsci.2011.04.002>

**Polyketide cyclase/dehydrase and lipid transport superfamily protein**

Quan W, Hu Y, Mu Z, Shi H, Chan Z (2018) Overexpression of AtPYL5 under the control of guard cell specific promoter improves drought stress tolerance in Arabidopsis. Plant Physiol Biochem 129: 150-157. doi: 10.1016/j.plaphy.2018.05.033

**PRA1 (Prenylated rab acceptor) family protein**

Pizarro L, Leibman-Markus M, Schuster S, Bar M, Meltz T, Avni A (2018) Tomato Prenylated RAB Acceptor Protein 1 modulates trafficking and degradation of the pattern recognition receptor LeEIX2, affecting the innate immune response. Front Plant Sci 9: 257. doi: 10.3389/fpls.2018.00257

**Programmed cell death protein**

NS Coll, P Epple, JL Dangl (2011) Programmed cell death in the plant immune system. Cell Death Diff 18: 1247–1256. doi:10.1038/cdd.2011.37

Locato V, De Gara L (2018) Programmed cell death in plants: an overview. Methods Mol Biol 1743: 1-8. doi: 10.1007/978-1-4939-7668-3_1

**Prohibitin**

Ruihua Huang, Chengwei Yang, Shengchun Zhang (2019) The Arabidopsis PHB3 is a pleiotropic regulator for plant development. Plant Signal Behav 14(11): e1656036. <https://doi.org/10.1080/15592324.2019.1656036>

**Proline-rich cell wall protein**

Bradley DJ, Kjellbom P, Lamb CJ (1992) Elicitor- and wound-induced oxidative cross-linking of a proline-rich plant cell wall protein: a novel, rapid defense response. Cell 70(1): 21-30.

**Prolyl 4-hydroxylase subunit alpha-1**

Kelly L Gorres, Ronald T Raines (2010) Prolyl 4-hydroxylase. Crit Rev Biochem Mol Biol 45(2): 106–124. doi:10.3109/10409231003627991

**Protease inhibitor/seed storage/lipid transfer family protein**

Jin-Young Kim, Seong-Cheol Park, Indeok Hwang, Hyeonsook Cheong, Jae-Woon Nah, Kyung-Soo Hahm, Yoonkyung Park (2009) Protease inhibitors from plants with antimicrobial activity. Int J Mol Sci 10: 2860-2872. doi:10.3390/ijms10062860

de Souza Cândido E, Pinto MFS, Pelegrini PB, Lima TB, Silva ON, Pogue R, Grossi-de-Sá MF, Franco OL (2011), Plant storage proteins with antimicrobial activity: novel insights into plant defense mechanisms. FASEB J 25: 3290-3305. <https://doi.org/10.1096/fj.11-184291>

Hang Gao, Kang Ma, Guojie Ji, Liying Pan, Qingfeng Zhou (2022) Lipid transfer proteins involved in plant–pathogen interactions and their molecular mechanisms. Mol Plant Pathol 23: 1815–1829. DOI: 10.1111/mpp.13264

**Ras-related protein Rab-18**

Manas K Tripathy, Renu Deswal, Sudhir K. Sopory (2021) Plant RABs: role in development and in abiotic and biotic stress responses Curr Genom 22: 26-40. DOI:10.2174/1389202922666210114102743

Vilardell J, Mundy J, Stilling B, Leroux B, Pla M, Freyssinet G, Pagès M. Regulation of the maize rab17 gene promoter in transgenic heterologous systems (1991) Plant Mol Biol 17(5): 985-993. doi: 10.1007/BF00037138

**Regulatory protein NPR1**

Corne MJ Pieterse, LC Van Loon (2004) NPR1: the spider in the web of induced resistance signaling pathways. Curr Opin Plant Biol 7: 456–464. DOI. 10.1016/j.pbi.2004.05.006

**Remorin**

Yu Y (2020) Remorins: essential regulators in plant-microbe interaction and cell death induction. Plant Physiol 183: 435-436.

**Respiratory burst oxidase**

**Reticuline oxidase-like protein**

Katarzyna Otulak-Koziel, Edmund Koziel, Rodrigo A Valverde (2019) The respiratory burst oxidase homolog D (RbohD) cell and tissue distribution in potato–Potato Virus Y (PVYNTN) hypersensitive and susceptible reactions. Int J Mol Sci Int 20: 2741; doi:10.3390/ijms20112741

Nobuhiro Suzuki, Gad Miller, Jorge Morales, Vladimir Shulaev, Miguel Angel Torres, Ron Mittler (2011) Respiratory burst oxidases: the engines of ROS signaling. Curr Opin Plant Biol 14: 691–699. DOI 10.1016/j.pbi.2011.07.014

**Response to salt stress**

Ma L, Liu X, Lv W and Yang Y (2022) Molecular mechanisms of plant responses to salt stress. Front Plant Sci 13: 934877. doi: 10.3389/fpls.2022.934877

**RGA2**

Loutre C, Wicker T, Travella S, Galli P, Scofield S, Fahima T, Feuillet C, Keller B (2009) Two different CC-NBS-LRR genes are required for Lr10-mediated leaf rust resistance in tetraploid and hexaploid wheat. Plant J 60(6): 1043-1054. doi: 10.1111/j.1365-313X.2009.04024.x.

**Root UVB sensitive 6**

# [Hongyun Tong](https://www.pnas.org/doi/full/10.1073/pnas.0809942106#con1), [Colin D Leasure](https://www.pnas.org/doi/full/10.1073/pnas.0809942106#con2), [Xuewen Hou](https://www.pnas.org/doi/full/10.1073/pnas.0809942106#con3), [Zheng-Hui He](https://www.pnas.org/doi/full/10.1073/pnas.0809942106#con6) (2008) Role of root UV-B sensing in *Arabidopsis* early seedling development. Proc Natl Acad Sci USA 105(52): 21039-21044. [www.pnas.org/cgi/doi/10.1073/pnas.0809942106](http://www.pnas.org/cgi/doi/10.1073/pnas.0809942106)

# Leasure CD, Tong H, Yuen G, Hou X, Sun X, He ZH (2009). ROOT UV-B SENSITIVE2 acts with ROOT UV-B SENSITIVE1 in a root ultraviolet B-sensing pathway. Plant Physiol 150(4): 1902-1915. doi: 10.1104/pp.109.139253.

# RP1-like

Wang X, Chen J, Yang Y, [Zhou](https://link.springer.com/article/10.1007/s11105-012-0537-0#auth-Jie-Zhou) J, [Qiu](https://link.springer.com/article/10.1007/s11105-012-0537-0#auth-Yan-Qiu) Y, [Yu](https://link.springer.com/article/10.1007/s11105-012-0537-0#auth-Chulang-Yu) C, [Cheng](https://link.springer.com/article/10.1007/s11105-012-0537-0#auth-Ye-Cheng) Y, [Yan](https://link.springer.com/article/10.1007/s11105-012-0537-0#auth-Chengqi-Yan) C, [Chen](https://link.springer.com/article/10.1007/s11105-012-0537-0#auth-Jianping-Chen) J (2013) Characterization of a novel NBS-LRR gene involved in bacterial blight resistance in rice. Plant Mol Biol Rep 31: 649–656. https://doi.org/10.1007/s11105-012-0537-0

**RPM1-interacting protein**

Zhao G, Guo D, Wang L, Li H, Wang C, Guo X (2021) Functions of RPM1-interacting protein 4 in plant immunity. Planta 253(1): 11. doi: 10.1007/s00425-020-03527-7

Sujit Kumar Ray, Donah Mary Macoy, Woe-Yeon Kim, Sang Yeol Lee, Min Gab Kim (2019) Role of RIN4 in regulating PAMP-triggered immunity and effector-triggered immunity: current status and future perspectives. Mol Cells 42(7): 503-511,

**S-formylglutathione hydrolase**

## S Kordic, Cummings I, Edwards R (2002) Cloning and characterization of an *S*-formylglutathione hydrolase from *Arabidopsis thaliana* [Arch Biochem Biophys](https://www.sciencedirect.com/journal/archives-of-biochemistry-and-biophysics) [399(2](https://www.sciencedirect.com/journal/archives-of-biochemistry-and-biophysics/vol/399/issue/2)): 232-238.

**S-norcoclaurine synthase**

**Pathogenesis-related (PR)-10-related norcoclaurine synthase-like protein**

Jillian M Hagel, Peter J Facchini (2013) Benzylisoquinoline alkaloid metabolism: a century of discovery and a brave new world. Plant Cell Physiol 54(5): 647–672. doi:10.1093/pcp/pct020

Hagel JM, Facchini PJ (2013) Benzylisoquinoline alkaloid metabolism: a century of

discovery and a brave new world. Plant Cell 22: 3489–3503. doi:10.1093/pcp/pct020

**SAGA-associated factor29**

Moraga F, Aquea F (2015) Composition of the SAGA complex in plants and its role in controlling gene expression in response to abiotic stresses. Front Plant Sci 6: 865. doi: 10.3389/fpls.2015.00865

**Salt stress root protein RS1**

Peter Nveawiah-Yoho, Jing Zhou, Marsha Palmer, Roger Sauve, Suping Zhou, Kevin J Howe, Tara Fish, Theodore W Thannhauser (2013) Identification of proteins for salt tolerance using a comparative proteomics analysis of tomato accessions with contrasting salt tolerance. J Am Soc Hort Sci 138(5): 382–394.

**Selenium-binding protein1**

## [Dutilleul C](https://www.uniprot.org/uniprotkb?query=lit_author:%22Dutilleul%20C.%22), [Jourdain A](https://www.uniprot.org/uniprotkb?query=lit_author:%22Jourdain%20A.%22), [Bourguignon J](https://www.uniprot.org/uniprotkb?query=lit_author:%22Bourguignon%20J.%22), [Hugouvieux V](https://www.uniprot.org/uniprotkb?query=lit_author:%22Hugouvieux%20V.%22) (2008) The Arabidopsis putative selenium-binding protein family: expression study and characterization of SBP1 as a potential new player in cadmium detoxification processes. Plant Physiol 147: 239–251.

[Hugouvieux V](https://www.uniprot.org/uniprotkb?query=lit_author:%22Hugouvieux%20V.%22), [Dutilleul C](https://www.uniprot.org/uniprotkb?query=lit_author:%22Dutilleul%20C.%22), [Jourdain A](https://www.uniprot.org/uniprotkb?query=lit_author:%22Jourdain%20A.%22), [Reynaud F](https://www.uniprot.org/uniprotkb?query=lit_author:%22Reynaud%20F.%22), [Lopez V](https://www.uniprot.org/uniprotkb?query=lit_author:%22Lopez%20V.%22), [Bourguignon J](https://www.uniprot.org/uniprotkb?query=lit_author:%22Bourguignon%20J.%22) (2009) Arabidopsis putative selenium-binding protein1 expression is tightly linked to cellular sulfur demand and can reduce sensitivity to stresses requiring glutathione for tolerance. Plant Physiol 151: 768-781.

**Senescence/dehydration-associated protein**

Barajas-Lopez JD, Tiwari A, Zarza X, Shaw MW, Pascual JS, Punkkinen M, Bakowska JC, Munnik T, Fujii H (2021) EARLY RESPONSE TO DEHYDRATION 7 remodels cell membrane lipid composition during cold stress in Arabidopsis. Plant Cell Physiol 62(1): 80-91. doi: 10.1093/pcp/pcaa139

**Serpin-ZX**

Harriet R Benbow, Lars S Jermiin, Fiona M Doohan (2019) Serpins: genome-wide characterisation and expression analysis of the serine protease inhibitor family in *Triticum aestivum*. G3 9: 2709.

**Silicon efflux transporter**

Nicolas Leroy, Félix de Tombeur, Yseult Walgraffe, Jean-Thomas Cornélis, François J Verheggen (2019) Silicon and plant natural defenses against insect pests: impact on plant volatile organic compounds and cascade effects on multitrophic interactions. Plants 8: 444. doi:10.3390/plants8110444

**SGT1**

Cristina Azevedo, Shigeyuki Betsuyaku, Jack Peart, Akira Takahashi , Laurent Noe, Ari Sadanandom, Catarina Casais, Jane Parker, Ken Shirasu (2006) Role of SGT1 in resistance protein accumulation in plant immunity. EMBO J 25: 2007–2016. doi:10.1038/sj.emboj.7601084

**SKP1-interacting protein 11**

Muhammad Naeem-ul-Hassan, Zamri Zainal, Chew Jin Kiat, Hossein Hosseini Monfared, Ismanizan Ismail (2017) *Arabidopsis thaliana* SKP1 interacting protein 11 (At2g02870) negatively regulates the release of green leaf volatiles. Royal Chem Soc Adv 7: 55725.

**Snake venom serine proteinase**

Member of CAP protein superfamily

Takashi Tadokoro, Cassandra M Modahl, Katsumi Maenaka, Narumi Aoki-Shioi (2020) Cysteine-rich secretory proteins (CRISPs) from venomous snakes: an overview of the functional diversity in a large and underappreciated superfamily. Toxins 12: 175. doi:10.3390/toxins12030175

**Soluble epoxide hydrolase**

**Epoxide hydrolase 2**

Gomi K, Yamamato H, Akimitsu K (2003) Epoxide hydrolase: a mRNA induced by the fungal pathogen *Alternaria alternata* on rough lemon (*Citrus jambhiri* Lush). Plant Mol Biol 53(1-2): 189-199. doi: 10.1023/B:PLAN.0000009287.95682.24

# Spermidine synthase

**Spermidine/putrescine import ATP-binding protein PotA**

# Seifi HA, Shelp BJ (2019) Spermine differentially refines plant defense responses against biotic and abiotic stresses. Front Plant Sci 10: 117. <https://doi.org/10.3389/fpls.2019.00117>

**SRC2**

Takahashi R, Shimosaka E (1997) cDNA sequence analysis and expression of two cold-regulated genes in soybean. Plant Sci 123(1-2): 93–104. https://doi.org/10.1016/S0168-9452(96)04568-2

**Stress-associated endoplasmic reticulum protein 2**

Manghwar H, Jianming Li J (2022) Endoplasmic reticulum stress and unfolded protein response

signaling in plants. Int J Mol Sci 23: 828. https://doi.org/10.3390/ijms23020828

Reyes-Impellizzeri S, Moreno AA (2021) The endoplasmic reticulum role in the plant response to abiotic stress. Front Plant Sci 12: 755447. doi: 10.3389/fpls.2021.755447

Yamaguchi A, Hori O, Stern DM, Hartmann E, Ogawa S, Tohyama M (1999) Stress-associated endoplasmic reticulum protein 1 (serp1)/ribosome-associated membrane protein 4 (ramp4) stabilizes membrane proteins during stress and facilitates subsequent glycosylation. J Cell Biol 147(6): 1195–1204.

**Stress inducible protein coi6.1**

No publication available

**Stress responsive A/B barrel domain**

Park SC, Lee JR, Shin SO, Park Y, Lee SY, Hahm KS (2007) Characterization of a heat-stable protein with antimicrobial activity from *Arabidopsis thaliana*. Biochem Biophys Res Commun 362(3): 562-567. doi: 10.1016/j.bbrc.2007.07.188

Shaik R, Ramakrishna W (2013) Genes and co-expression modules common to drought and bacterial stress responses in *Arabidopsis* and rice. PLoS ONE 8(10): e77261. doi:10.1371/journal.pone.0077261

**Stress-induced phosphoprotein 1**

Toribio R, Mangano S, Fernández-Bautista N, Muñoz A, Castellano MM (2020) HOP,

a co-chaperone involved in response to stress in plants. Front Plant Sci 11: 591940. doi: 10.3389/fpls.2020.591940

**Strictosidine synthase-like**

Ting Zou, Shuangcheng Li, Mingxing Liu, Tao Wang, Qiao Xiao, Dan Chen, Qiao Li, Yanling Liang, Jun Zhu, Yueyang Liang, Qiming Deng, Shiquan Wang, Aiping Zheng, Lingxia Wang, Ping Li (2017) An atypical strictosidine synthase, OsSTRL2, plays key roles in anther development and pollen wall formation in rice. Nature Sci Rep 7: 6863. DOI:10.1038/s41598-017-07064-4

**Sucrose nonfermenting 4-like**

Zhiwei Chen, Longhua Zhou, Panpan Jiang, Ruiju Lu, Nigel G Halford, Chenghong Liu (2021) Genome-wide identification of sucrose nonfermenting-1-related protein kinase (SnRK) genes in barley and RNA-seq analyses of their expression in response to abscisic acid treatment. BMC Genom 22: 300. https://doi.org/10.1186/s12864-021-07601-6

**Sucrose phosphate synthase**

**Sucrose-phosphatase**

**Sucrose synthase**

Poonam Kanwar, Gopaljee Jha (2019) Alterations in plant sugar metabolism: signatory of pathogen attack. Planta 249(2): 305-318. <https://doi.org/10.1007/s00425-018-3018-3>

Liu YH, Song YH, Ruan YL (2022) Sugar conundrum in plant-pathogen interactions: roles of invertase and sugar transporters depend on pathosystems. J Exp Bot 73(7): 1910-1925. doi: 10.1093/jxb/erab562.

**Sucrose-proton symporter**

**Sugar transport protein/sugar transporter**

Breia R, Conde A, Badim H, Fortes AM, Gerós H, Granell A (2021) Plant SWEETs: from sugar transport to plant-pathogen interaction and more unexpected physiological roles. Plant Physiol 186(2): 836-852. doi: 10.1093/plphys/kiab127

Weilong Kong, Baoguang An, Yue Zhang, Jing Yang, Shuangmiao Li, Tong Sun, Yangsheng Li (2019) Sugar transporter proteins (STPs) in Gramineae crops: comparative analysis, phylogeny, evolution, and expression profiling. Cells 8: 560. doi:10.3390/cells8060560

**Sugar transporter ERD6-like**

Slawinski L, Israel A, Artault C, Thibault F, Atanassova R, Laloi M, Dédaldéchamp F (2021)

Responsiveness of early response to Dehydration Six-like transporter genes to water deficit in *Arabidopsis thaliana* leaves. Front. Plant Sc. 12: 708876. doi: 10.3389/fpls.2021.708876

**Superoxide dismutase**

Raychaudhuri S, Deng XW (2000) The role of superoxide dismutase in combating oxidative stress in higher plants. Bot. Rev 66: 89–98. <https://doi.org/10.1007/BF02857783>

Navrot N, Routhier N, Gelhaye E, Jacquot J-P (2007) Reactive oxygen species generation and antioxidant systems in plant mitochondria. Physiol Plantar 129: 185–195. <https://doi.org/10.1111/j.1399-3054.2006.00777.x>

**Taxadiene synthase**

Mustafa Köksal, Yinghua Jin, Robert M Coates, Rodney Croteau, David W Christianson (2011) Taxadiene synthase structure and evolution of modular architecture in terpene biosynthesis. Nature 469(7328): 116–120. doi:10.1038/nature09628

**Terpene synthase**

Ai-Xia Cheng, Yong-Gen Lou, Ying-Bo Mao, Shan Lu, Ling-Jian Wang, Xiao-Ya Chen (2007) Plant terpenoids: biosynthesis and ecological functions. J Integr Plant Biol 49(2): 179−186.

**Taxol biosynthesis**

# [Da Cheng Hao](https://link.springer.com/article/10.1007/s10709-008-9257-7#auth-Da_Cheng-Hao), [Ling Yang](https://link.springer.com/article/10.1007/s10709-008-9257-7#auth-Ling-Yang), [Beili Huang](https://link.springer.com/article/10.1007/s10709-008-9257-7#auth-Beili-Huang) (2009) Molecular evolution of paclitaxel biosynthetic genes *TS* and *DBAT* of *Taxus* species. [Genetica](https://link.springer.com/journal/10709) 135: 123–135.

**Tetraspanin family protein**

Saul Jimenez-Jimenez, Kenji Hashimoto, Olivia Santana, Jesús Aguirre, Kazuyuki Kuchitsu, Luis Cárdenas (2019) Emerging roles of tetraspanins in plant inter-cellular and inter-kingdom communication. Plant Signal Behav 14(4): e1581559. https://doi.org/10.1080/15592324.2019.1581559

**Thionin**

Hong K, Austerlitz T, Bohlmann T, Bohlmann H (2021) The thionin family of antimicrobial peptides. PLoS ONE 16(7): e0254549. https://doi.org/10.1371/journal.pone.0254549

**Thioredoxin**

**Thioredoxin reductase**

Christina Vieira Dos Santos, Pascal Rey (2006) Plant thioredoxins are key actors in the oxidative stress response. Trends Plant Sci 11(7): 329-334. <https://doi.org/10.1016/j.tplants.2006.05.005>

**Thiosulfate sulfurtransferase**

Most P, Papenbrock J (2015) Possible roles of plant sulfurtransferases in detoxification of cyanide, reactive oxygen species, selected heavy metals and arsenate. Molecules 20: 1410-1423. doi:10.3390/molecules20011410

**TMV-MP30 binding protein**

Florian Vogel, Daniel Hofius,Uwe Sonnewald (2007) Intracellular trafficking *of Potato Leafroll Virus* movement protein in transgenic *Arabidopsis*. Traffic 8: 1205–1214. doi: 10.1111/j.1600-0854.2007.00608.x

# Tobamovirus multiplication protein

Qun Hu, Hui Zhang, Lei Zhang, Yong Liu, Changjun Huang, Cheng Yuan, Zefan Chen, Kunpeng Li, Robert M Larkin, Jiongjiong Chen, Hanhui Kuang (2021) Two TOBAMOVIRUS MULTIPLICATION 2A homologs in tobacco control asymptomatic response to tobacco mosaic virus. Plant Physiol 87: 2674–2690. doi:10.1093/plphys/kiab448

# TOM1-like protein

Takuya Yamanaka, Takehiro Ohta, Miki Takahashi, Tetsuo Meshi, Renate Schmidt Caroline Dean, Satoshi Naito, Masayuki Ishikawa (2000) TOM1, an Arabidopsis gene required for efficient multiplication of a tobamovirus, encodes a putative transmembrane protein. Proc Natl Acad Sci USA 97(18): 10107–10112 .

**Binding to TOMV RNA 1L**

**TOM1 RNA binding protein**

Fujisaki K, Ishikawa M (2008) Identification of an *Arabidopsi*s thaliana protein that binds to tomato mosaic virus genomic RNA and inhibits its multiplication. Virology 380(2): 402-411. doi: 10.1016/j.virol.2008.07.033

**Transthyretin-like S-allantoin synthase**

Tao Chen, Wei Zhang, Gang Yang Jia-Hui, Chen, Bi-Xia Chen, Rui Sun, Hua Zhang, Li-Zhe An (2020) TRANSTHYRETIN-LIKE and BYPASS1-LIKE coregulate growth and cold tolerance in Arabidopsis Plant Biol 20: 332. https://doi.org/10.1186/s12870-020-02534-w

**Trehalose phosphate synthase**

Li HW, Zang BS, Deng XW, Wang XP (2011) Overexpression of the trehalose-6-phosphate synthase gene OsTPS1 enhances abiotic stress tolerance in rice. Planta. 234(5): 1007-1018. doi: 10.1007/s00425-011-1458-0

Gabriel Iturriaga, Ramón Suárez, Barbara Nova-Franco (2009) Trehalose metabolism: from osmoprotection to signaling. Int J Mol Sci 10: 3793-3810. doi:10.3390/ijms10093793

**Trichome birefringence-like**

Haris Ahmed, Mian Faisal Nazir, Zhoe Pan, Wenfang Gong, Muhammad Shahid Iqbal, Shoupu He, Xiongming Du (2020). Genotyping by sequencing revealed QTL hotspots for trichome-based plant defense in *Gossypium hirsutum*. Genes 11: 368. doi:10.3390/genes11040368

**Tropinone reductase**

Divekar PA, Narayana S, Divekar BA, Kumar R, Gadratagi BG, Ray A, Singh AK, Rani V, Singh V, Singh AK, Kumar A, Singh RP, Meena RS, Behera TK (2022) Plant secondary metabolites as defense tools against herbivores for sustainable crop protection. Int J Mol Sci 23(5): 2690. doi: 10.3390/ijms23052690

**Trypsin inhibitor**

**Trypsin proteinase inhibitor**

Jorge A Zavala, Aparna G Patankar, Klaus Gase, Dequan Hui, Ian T Baldwin (2004) Manipulation of endogenous trypsin proteinase inhibitor production in *Nicotiana attenuata* demonstrates their function as antiherbivore defenses. Plant Physiol 134: 1181–1190. [www.plantphysiol.org/cgi/doi/10.1104/pp.103.035634](http://www.plantphysiol.org/cgi/doi/10.1104/pp.103.035634)

**UNC93-like protein**

**Undecaprenyl-phosphate 4-deoxy-4-formamido-L-arabinose transferase**

Xiang J, Zhou X, Zhang X, Liu A, Xiang Y, Yan M, Peng Y, Chen X (2018) The Arabidopsis AtUNC-93 acts as a positive regulator of abiotic stress tolerance and plant growth via modulation of ABA signaling and K+ homeostasis. Front Plant Sci 9: 718. doi: 10.3389/fpls.2018.00718

Breazeale SD, Ribeiro AA, Raetz CR (2002) Oxidative decarboxylation of UDP-glucuronic acid in extracts of polymyxin-resistant *Escherichia coli*. Origin of lipid a species modified with 4-amino-4-deoxy-L-arabinose. J Biol Chem 277(4): 2886-2896. doi: 10.1074/jbc.M109377200

**Universal stress protein**

Chi YH, Koo SS, Oh HT, Lee ES, Park JH, Phan KAT, Wi SD, Bae SB, Paeng SK, Chae HB, Kang CH, Kim MG, Kim W-Y, Yun D-J, Lee SY (2019) The physiological functions of universal stress proteins and their molecular mechanism to protect plants from environmental stresses. Front Plant Sci 10: 750. doi: 10.3389/fpls.2019.00750

**UV excision repair protein rad23**

Triparna Lahari, Janelle Lazaro, Dana F Schroeder (2018) RAD4 and RAD23/HMR contribute to Arabidopsis UV tolerance. Genes 9: 8. doi:10.3390/genes9010008

**Very-long-chain enoyl-CoA reductase**

Wang J, Chen Y-L, Li Y-K, Chen D-K, He J-F and Yao N (2021) Functions of sphingolipids in pathogenesis during host–pathogen interactions. Front Microbio 12: 701041. doi: 10.3389/fmicb.2021.701041

**VIP1**

Tzvi Tzifra, Manjusha Vaidya, Vitaly Citovsky (2001) VIP1, an *Arabidopsis* protein that interacts with *Agrobacterium* VirE2, is involved in VirE2 nuclear import and *Agrobacterium* infectivity. EMBO J 20(13): 3596-3607.

**Wall-associated receptor kinase**

Bruce D Kohorn, Susan L Kohorn (2012) The cell wall-associated kinases, WAKs, as pectin receptors. Front Plant Sci 3: 88. doi: 10.3389/fpls.2012.00088

**Wound-induced protein 1**

Logemann J, Schell J (1989) Nucleotide sequence and regulated expression of a wound-inducible potato gene (wun1). Mol Gen Genet 219(1-2): 81-88. doi: 10.1007/BF00261161

Daniel Savatin, Giovanna Gramegna, Vanessa Modesti, Felice Cervone (2014) Wounding in the plant tissue: the defense of a dangerous passage. Front Plant Sci 5: 470. doi: 10.3389/fpls.2014.00470

**Wound-induced protease inhibitor**

Hugo Pena-Cortes, Joachim Fisahn, Lothar Willmitzer (1995) Signals involved in wound-induced proteinase inhibitor II gene expression in tomato and potato plants. Proc Natl Acad Sci USA 92: 4106-4113.

**Wound-responsive family protein**

UniProt A0A438DQF0_VITVI

**WRKY DNA-binding protein 33**

Wani SH, Anand S, Singh B,  [Bohra](https://link.springer.com/article/10.1007/s00299-021-02691-8#auth-Abhishek-Bohra) A, [Joshi](javascript:;) R (2021) WRKY transcription factors and plant defense responses: latest discoveries and future prospects. Plant Cell Rep 40: 1071–1085. https://doi.org/10.1007/s00299-021-02691-8

**Xyloglucan endotransglucosylase/hydrolase**

Seok Keun Cho, Jee Eun Kim, Jong-A Park, Tae Jin Eom, Woo Taek Kim (2006) Constitutive expression of abiotic stress-inducible hot pepper CaXTH3, which encodes a xyloglucan endotransglucosylase/hydrolase homolog, improves drought and salt tolerance in transgenic Arabidopsis plants. FEBS Lett 580: 3136–3144.

**YTH domain proteins**

Weiwei Ma, Song Cui, Zhenfei Lu, Xiaofeng Yan, Long Cai, Yongfa Lu, Kefeng Cai, Huacheng Zhou, Rongrong Ma, Shirong Zhou, Xiaole Wang (2022) YTH domain proteins play an essential role in rice growth and stress response. Plants 11(17): 2206. <https://doi.org/10.3390/plants11172206>

**Zinc finger proteins**

**A20/AN1 zinc-finger**

[Shubha Vij](https://link.springer.com/article/10.1007/s00438-006-0165-1#auth-Shubha-Vij), [Akhilesh K. Tyagi](https://link.springer.com/article/10.1007/s00438-006-0165-1#auth-Akhilesh_K_-Tyagi) (2006) Genome-wide analysis of the stress associated protein (SAP) gene family containing A20/AN1 zinc-finger(s) in rice and their phylogenetic relationship with *Arabidopsis*. Mol Genet Genom 276: 565–575.

**C_2_H_2_ type**

## Agnieszka Kiełbowicz-Matuk (2012) Involvement of plant C_2_H_2_-type zinc finger transcription factors in stress responses**.** Plant Sci [185–186](https://www.sciencedirect.com/journal/plant-science/vol/185/suppl/C): 78-85. <https://doi.org/10.1016/j.plantsci.2011.11.015>

**CCCH domain**

Guoliang Han, Ziqi Qiao, Yuxia Li, Chengfeng Wang, Baoshan Wang (2021) The roles of CCCH zinc-finger proteins in plant abiotic stress tolerance. Int J Mol Sci. 22: 8327. <https://doi.org/10.3390/ijms22158327>

**C3HC4 RING-finger**

Preeti Agarwal, Paramjit Khurana (2018) Characterization of a novel zinc finger transcription factor (TaZnF) from wheat conferring heat stress tolerance in Arabidopsis. Cell Stress Chaperones 23: 253–267. DOI 10.1007/s12192-017-0838-1

**CONSTANS-like**

**CONSTANS**

# [Ji-Hee Min](https://onlinelibrary.wiley.com/authored-by/Min/Ji%E2%80%90Hee), [Jung-Sung Chung](https://onlinelibrary.wiley.com/authored-by/Chung/Jung%E2%80%90Sung), [Kyeong-Hwan Lee](https://onlinelibrary.wiley.com/authored-by/Lee/Kyeong%E2%80%90Hwan), [Cheol Soo Kim](https://onlinelibrary.wiley.com/authored-by/Kim/Cheol+Soo) (2014) The CONSTANS-like 4 transcription factor, AtCOL4, positively regulates abiotic stress tolerance through an abscisic acid-dependent manner in *Arabidopsis.* J Integ Plant Biol 57: 313–324. doi: 10.1111/jipb.12246

**FYVE-domain**

[Shanshan Xiao](https://link.springer.com/article/10.1007/s11105-016-0988-9#auth-Shanshan-Xiao), [Min Shao](https://link.springer.com/article/10.1007/s11105-016-0988-9#auth-Min-Shao), [Dong Wang](https://link.springer.com/article/10.1007/s11105-016-0988-9#auth-Dong-Wang), [Wenqi Li](https://link.springer.com/article/10.1007/s11105-016-0988-9#auth-Wenqi-Li), [Fengquan Liu](https://link.springer.com/article/10.1007/s11105-016-0988-9#auth-Fengquan-Liu) (2016) Identification and evolution of FYVE domain-containing proteins and their expression patterns in response to abiotic stresses in rice. Plant Mol Biol Rep 34: 1064–1082.

**Zinc‐binding dehydrogenase**

# Dilip Kumar, Sakshi Rampuria, Naveen Kumar Singh, Pulugurtha B. Kirti (2016) A novel zinc‐binding alcohol dehydrogenase 2 from Arachis diogoi, expressed in resistance responses against late leaf spot pathogen, induces cell death when transexpressed in tobacco. FEBS Open Bio 6: 200–210. doi:10.1002/2211-5463.12040

**Disease resistance proteins**

**CC-NBS-LRR class**

**NBS-LRR**

**TIR-NBS**

**TIR-NBS-LRR class**

**RPM1**

**RPM3**

**RPP13**

Martin GB, Bogdanove AJ, Sessa G (2003) Understanding the functions of plant disease resistance proteins. Annu Rev Plant Biol 54: 23-61. doi: 10.1146/annurev.arplant.54.031902.135035

Plant disease resistance protein signaling: NBS–LRR proteins and their partners

Youssef Belkhadir, Rajagopal Subramaniam, Jeffery L Dangl (2004) Curr Opin Plant Biol 7: 391–399. DOI 10.1016/j.pbi.2004.05.009

**Pathogenesis-related proteins**

**beta-1,3-glucanase**

**Chitinase**

**Endochitinase**

**Endoglucanase**

**Glucan 1,3-beta-glucosidase**

**Glucan endo-1,3-beta-glucosidase-like protein 3**

**Pathogen-related protein 1**

**Pathogenesis-related protein 1**

**Pathogenesis-related thaumatin superfamily protein**

Linthorst HJM, [Van Loon LC](https://www.tandfonline.com/author/van+Loon%2C+LC+PhD) (1991) Pathogenesis-related proteins of plants. Crit Rev Plant Sci 10(2): 123-150. <https://doi.org/10.1080/07352689109382309>

**Oxidative stress metabolism**

**1-Cys peroxiredoxin A**

**Ascorbate peroxidase**

**Catalase**

**Catalase-peroxidase**

**Fe superoxide dismutase**

**Glutaredoxin**

**Glutathione peroxidase**

**Glutathione reductase**

**Glutathione S-transferase**

**Microsomal glutathione S-transferase 3**

**Glutathione synthetase**

**Monodehydroascorbate reductase**

Kaushik Das, Aryadeep Roychoudhury (2014) Reactive oxygen species (ROS) and response of antioxidants as ROS-scavengers during environmental stress in plants. Front Environ Sci 2: 53. doi: 10.3389/fenvs.2014.00053

Navrot N, Routhier N, Gelhaye E, Jacquot J-P (2007) Reactive oxygen species generation and antioxidant systems in plant mitochondria. Physiol Plantar 129: 185–195. <https://doi.org/10.1111/j.1399-3054.2006.00777.x>

**Sterol biosynthesis**

**3-beta-hydroxysteroid dehydrogenase/Delta 5-->4-isomerase type 3**

# 3-beta-hydroxysteroid-Delta(8),Delta(7)-isomerase

**C-5 sterol desaturase & C-8,7 sterol isomerase***

**Delta-sterol C5(6)-desaturase**

**Delta(24)-sterol reductase***

**Delta(24)-sterol reductase**

**Ergosterol biosynthetic protein 28***

**Ergosterol biosynthetic protein 28**

**Methylsterol monooxygenase 2-1**

**Methylsterol monooxygenase***

**Squalene synthase***

**Squalene synthase**

**Sterol 3-beta-glucosyltransferase**

**Sterol C4-methyl oxidase**

**Sterol methyltransferase**

**Delta(7)-sterol-C5(6)-desaturase**

**Squalene monooxygenase**

**Steroid 5-alpha reductase**

**Sterol-8,7-isomerase**

Keri Wang, Muthappa Senthil-Kumar, Choong-Min Ryu, Li Kang, Kirankumar S Mysore (2012) Phytosterols play a key role in plant innate immunity against bacterial pathogens by regulating nutrient efflux into the apoplast. Plant Physiol 158: 1789–1802.

[Yinglin Du](https://sciprofiles.com/profile/1932088), [Xizhe Fu](https://sciprofiles.com/profile/author/SC9QaGptYlJYR1RpRlBqRE5hRi9UKzhUb1I0M0o2UWJ5RnpheVlmZ3l1Yz0=), [Yiyang Chu](https://sciprofiles.com/profile/author/NUxnQWEvbWh4V3Rsa3JMWTl2eEZ3QlQzQXVrTDNobmQ3cHFKVTVKYW8yZz0=), [Peiwen Wu](https://sciprofiles.com/profile/author/MU44NGlsVHlnUE1HMGVQK1JpdzhtQT09), [Ye Liu](https://sciprofiles.com/profile/author/UmlMOFR1eENBMi85akU3bW1FWGRKcGtkWCs1c0t0M0ZQTy80cnVZZERBRT0=), [Lili Ma](https://sciprofiles.com/profile/author/ajkzcFoweEp5M2piUUxud3p5SjBWS3Zleldnb2tRMU13SGxDS2N5OFExZz0=), [Huiqin Tian](https://sciprofiles.com/profile/author/YnAvcXhEcW9yQlFhbmZLYXNJUkVRcjdZRFlWd2c1aHNDTHRNWGphT0x2ST0=), [Benzhong Zhu](https://sciprofiles.com/profile/2009386) (2022) Biosynthesis and the roles of plant sterols in development and stress responses. Int J Mol Sci 23(4): 2332. <https://doi.org/10.3390/ijms23042332>
